# Glucocorticoids enhance chemotherapy-driven stress granule assembly and impair granule dynamics, leading to cell death

**DOI:** 10.1242/jcs.259629

**Published:** 2022-07-26

**Authors:** Avital Schwed-Gross, Hila Hamiel, Gabriel P. Faber, Mor Angel, Rakefet Ben-Yishay, Jennifer I. C. Benichou, Dana Ishay-Ronen, Yaron Shav-Tal

**Affiliations:** 1 The Mina and Everard Goodman Faculty of Life Sciences, Bar-Ilan University, Ramat Gan 5290002, Israel; 2 Institute of Nanotechnology, Bar-Ilan University, Ramat Gan 5290002, Israel; 3 Oncology Institute, Chaim Sheba Medical Center, Tel-Hashomer, Ramat Gan 52621, Israel

**Keywords:** Stress granules, Cortisone, Vinorelbine, Translation, EIF2α, Cell death, FRAP

## Abstract

Stress granules (SGs) can assemble in cancer cells upon chemotoxic stress. Glucocorticoids function during stress responses and are administered with chemotherapies. The roles of glucocorticoids in SG assembly and disassembly pathways are unknown. We examined whether combining glucocorticoids such as cortisone with chemotherapies from the vinca alkaloid family, which dismantle the microtubule network, affects SG assembly and disassembly pathways and influences cell viability in cancer cells and human-derived organoids. Cortisone augmented SG formation when combined with vinorelbine (VRB). Live-cell imaging showed that cortisone increased SG assembly rates but reduced SG clearance rates after stress, by increasing protein residence times within the SGs. Mechanistically, VRB and cortisone signaled through the integrated stress response mediated by eIF2α (also known as EIF2S1), yet induced different kinases, with cortisone activating the GCN2 kinase (also known as EIF2AK4). Cortisone increased VRB-induced cell death and reduced the population of cells trapped in mitotic catastrophe. These effects were mediated by the core SG proteins G3BP1 and G3BP2. In conclusion, glucocorticoids induce SG assembly and cell death when administered with chemotherapies, suggesting that combining glucocorticoids with chemotherapies can enhance cancer cell chemosensitivity.

## Abstract (precis)

**Summary:** Combining cortisone with the chemotherapy vinorelbine enhances the assembly of stress granules that are less likely to be cleared from the cells, augmenting vinorelbine-induced cell death.

## INTRODUCTION

Cells contain a variety of granules, which are non-membrane-bound structures, that harbor various RNA-binding proteins (RBPs) and RNAs. Cytoplasmic granules include P bodies (PBs), stress granules (SGs), germ cell granules, neuronal granules and others (Buchan, 2014; Erickson and Lykke-Andersen, 2011; Riggs et al., 2020). Some granule types occur naturally in cells, such as PBs, whereas some can be induced. The assembly of cytoplasmic SGs occurs when cells are exposed to a diverse array of stresses, such as oxidative stress, hypoxia, heat shock, nutrient deprivation, viral infection or chemotoxic stress (Anderson et al., 2015; Kedersha et al., 2013; Nover et al., 1989; Protter and Parker, 2016; Zeng et al., 2020).

SGs are irregular structures that can vary in number per cell and in size (Anderson and Kedersha, 2009; Jain et al., 2016; Moser and Fritzler, 2010). While their exact functions are yet to be elucidated (Kedersha et al., 2013; Mateju and Chao, 2021), it is clear that they harbor unique protein and RNA compositions that distinguish them from related granules such as PBs. SGs typically contain untranslated mRNAs, but also microRNAs and long non-coding RNAs (lncRNAs) (Bounedjah et al., 2014; Campos-Melo et al., 2021; Khong et al., 2017; Lavut and Raveh, 2012). It has been suggested that during stress, certain mRNAs, proteins and parts of the translation machinery localize to SGs in order to protect vital cellular components. A large variety of RBPs are found within SGs (Jain et al., 2016; Youn et al., 2019); these normally take part in various cellular processes including mRNA transcription, splicing and export, as well as RNA stability and translation regulation (Sharma et al., 2021). Some proteins are considered hallmark SG markers, such as G3BP1 and G3BP2 (referred to collectively as G3BP1/2), TIA-1, TIAR (also known as TIAL1) and IGF2BP proteins (Stohr et al., 2006; Tourriere et al., 2003; van Leeuwen and Rabouille, 2019). Components of the 40S ribosomal subunit, together with some translation initiation factors, are found within SGs due to ribosome runoff. These might be in waiting for return of stalled mRNAs back to the active translation machinery when the stress signals dissipate (Advani and Ivanov, 2019; Protter and Parker, 2016).

Two signaling pathways inhibit the translation machinery during cellular stress and can drive SG assembly (Jackson et al., 2010; Wolozin and Ivanov, 2019). One acts through the inhibition of eIF4F complex assembly (Emara et al., 2012; Fujimura et al., 2012; Sonenberg and Hinnebusch, 2009), while the other asserts its action by phosphorylating the translation factor eIF2 on its α subunit (also known as EIF2S1) at the serine 51 position. eIF2 is part of the translation pre-initiation complex. Phosphorylation of eIF2α leads to strong association with eIF2B (also known as EIF2S2), a guanine-nucleotide-exchange factor (GEF), which in turn prevents formation of the eIF2–GTP–tRNA^Met^ ternary complex required for translation initiation. Altogether, this prevents assembly of the 48S pre–initiation complex, halting translation (Merrick and Pavitt, 2018; Panas et al., 2016). This is termed the integrated stress response (ISR) (Wek, 2018). Four kinases are responsible for the phosphorylation of eIF2α in mammalian cells, each prompted by different stresses (Aulas et al., 2017; Wolozin and Ivanov, 2019). PERK [protein kinase R (PKR)-like endoplasmic reticulum (ER) kinase, also known as EIF2AK3] is induced by ER stress (Harding et al., 2000). HRI (heme-regulated inhibitor, also known as EIF2AK1) is stimulated by oxidative stress, heat shock or mitochondrial stress (Girardin et al., 2021; McEwen et al., 2005). GCN2 (general control non-derepressible protein 2, also known as EIF2AK4) is activated by amino acid starvation (Wek et al., 1995). Finally, PKR kinase (also known as EIF2AK2) is triggered by double-stranded RNA upon RNA virus infection (García et al., 2007).

The connection between SG formation and cancer is well documented (Anderson et al., 2015). Upregulation in the expression of certain SG factors has been connected to tumor development and metastasis, as well as conveying resistance to chemotherapeutics. SGs are connected to other disease states such as the etiology of neurodegenerative diseases (Adjibade et al., 2015; Grabocka and Bar-Sagi, 2016; Mahboubi and Stochaj, 2017; Marmor-Kollet et al., 2020; Wang et al., 2018; Wolozin and Ivanov, 2019). Indeed, oxidative stress is one of the characteristics of the tumor microenvironment and, hence, might support the induction of SG assembly and promote cell survival (Arimoto et al., 2008; Thedieck et al., 2013).

Drugs that disrupt the microtubule network play a crucial role in cancer treatment by interfering with cancer cell proliferation, and they also affect SG formation (Franchini et al., 2019; Parker et al., 2014; Szaflarski et al., 2016). Microtubule-targeting agents have been shown to inhibit SG assembly, whereas microtubule-stabilizing drugs have the opposite effect (Bartoli et al., 2011; Chernov et al., 2009; Ivanov et al., 2003; Nadezhdina et al., 2010; Szaflarski et al., 2016; Wang et al., 2020). Vinca alkaloids are cytotoxic, anti-mitotic, microtubule-destabilizing drugs that are in clinical use for treatment of variety of cancers, and they include vinorelbine (VRB) and vinblastine (VBL) (Moudi et al., 2013). They have been particularly effective in treating women with metastatic breast cancers and patients with non-small cell lung cancer and metastatic sarcoma (Degardin et al., 1994; Nobili et al., 2020; Piccirillo et al., 2010; Vogel et al., 1999). Currently, oral VRB is administered to patients as a metronomic chemotherapy for both breast and non-small cell lung cancers (Liu et al., 2021; Xu et al., 2021). Vinca alkaloids promote translation repression and assembly of SGs by signaling through the ISR (Szaflarski et al., 2016). Cells lacking core SG proteins important for SG assembly are more vulnerable to cell death driven by the vinca alkaloids (Fujimura et al., 2012; Szaflarski et al., 2016).

It is common to administer glucocorticoids to cancer patients receiving chemotherapy. Glucocorticoids are hormones that play a role in the stress response and have anti-inflammatory properties, and therefore they are used to treat inflammatory disease (Herr et al., 2003). Glucocorticoids can cause oxidative stress (Almeida et al., 2011) and are connected to cell death by inducing apoptosis (Gruver-Yates and Cidlowski, 2013; Kofler, 2000; Planey and Litwack, 2000). The latter, together with the severe side effects of chemotherapeutic drugs, has led to frequent cotreatment of cancer patients with glucocorticoids and chemotherapy. In this study, we examined whether the addition of glucocorticoids, specifically cortisone, to cells treated with the vinca alkaloid VRB would modulate different properties of SG formation induced by VRB. By studying SG dynamics, the ISR signal transduction pathways, cell viability and cell death pathways, we conclude that when cortisone is combined with VRB it impairs the biophysical properties of the SG assembly and disassembly processes, having an enhancing and prolonged effect on the kinetics of VRB-induced SG formation, thereby increasing the sensitivity of cancer cells to chemotherapy.

## RESULTS

### Cortisone enhances the formation of SGs under VRB stress conditions

**Fig. 1.**
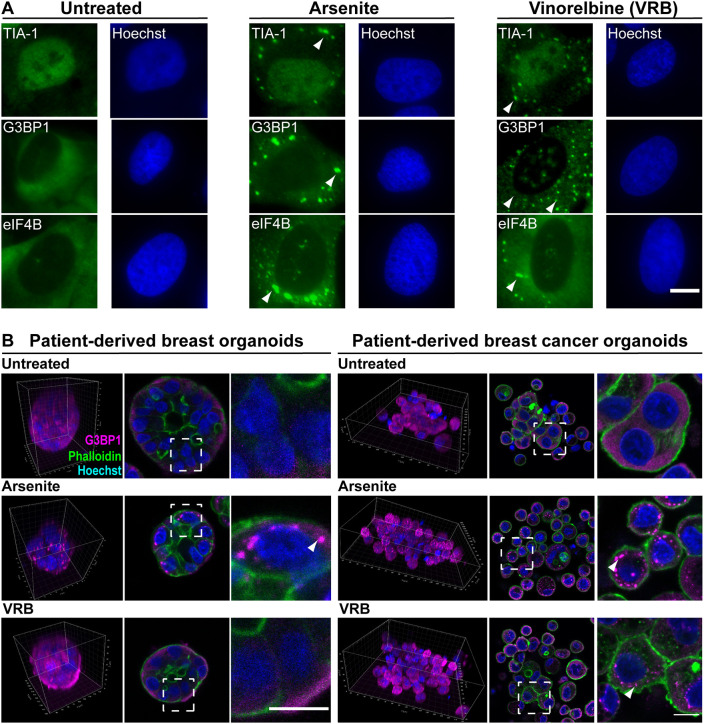
**VRB induces SG formation in cells and organoids.** (A) SG markers TIA-1, G3BP1 and eIF4B (green) were detected in untreated, arsenite-treated (45 min, 0.25 mM) and VRB-treated (1 h, 75 µM) U2OS cells by immunofluorescence. Hoechst 33342 DNA stain is shown in blue. Arrowheads mark some SGs. Scale bar: 10 µm. (B) SG formation was examined in patient-derived breast organoids originating from either healthy tissue or from cancerous tissue. Organoids were treated with arsenite (1 h, 0.25 mM) or VRB (2 h, 75 μM). SGs were detected with anti-G3BP1 antibody (magenta). Phalloidin staining of actin (green) was used to detect the cell outline. Hoechst 33342 DNA stain is shown in blue. Images on the left show 3D presentations of the organoids. Images in the middle show organoid sections, with dashed boxes indicating regions shown in the enlargements on the right. Arrowheads mark SGs. Scale bars: 10 µm. Images in A and B are representative of three experiments.

VRB is used for the treatment of breast cancer and non-small cell lung cancer. It targets the microtubule network and prevents spindle formation in cancer cells, hence disrupting cell division and proliferation. VRB and other vinca alkaloids, when administered to cultured cancer cell lines, induce the formation of SGs and influence cell survival (Szaflarski et al., 2016). Many cancer patients receive steroid hormone treatments in addition to chemotherapy. While some steroid hormones have been shown to reduce SG formation in HeLa cells (Timalsina et al., 2018), the effect of glucocorticoids in SG formation has not been examined. Therefore, we tested the effect of cortisone on SG formation, SG dynamics, signal transduction pathways and cell survival. We first verified that the VRB treatment (75 µM) induced SG formation. Indeed, SGs appeared in human osteosarcoma-derived U2OS cells 1 h following VRB administration and were detected by the presence of known SG components (Fig. 1A). Cells under oxidative stress induced by arsenite were used as a positive control for SG assembly.

Organoids are three-dimensional (3D) cell cultures that can mimic tissue architecture and can be generated from human tissues (Kim et al., 2020). Organoids form hollow or budding spheres, representing multiple cell populations from a tumor. SGs have been identified in induced pluripotent stem cell (iPSC)-derived cerebral organoids (Bowles et al., 2021; Zhao et al., 2020) but not in cancer-derived organoids. We tested the formation of SGs in the presence of VRB and arsenite in organoids formed from patient-derived healthy mammary gland tissue (BR33N) and from breast cancer tissue (HUB-01-C2-152), the latter originating from metastatic tissue (Sachs et al., 2018) (Fig. 1B; Fig. S1). Whereas arsenite induced SGs in both types of organoids, no SGs were detected during VRB treatment of organoids generated from the patient-derived normal breast tissue, and SGs only formed in the organoids from the breast cancer tissue.

**Fig. 2.**
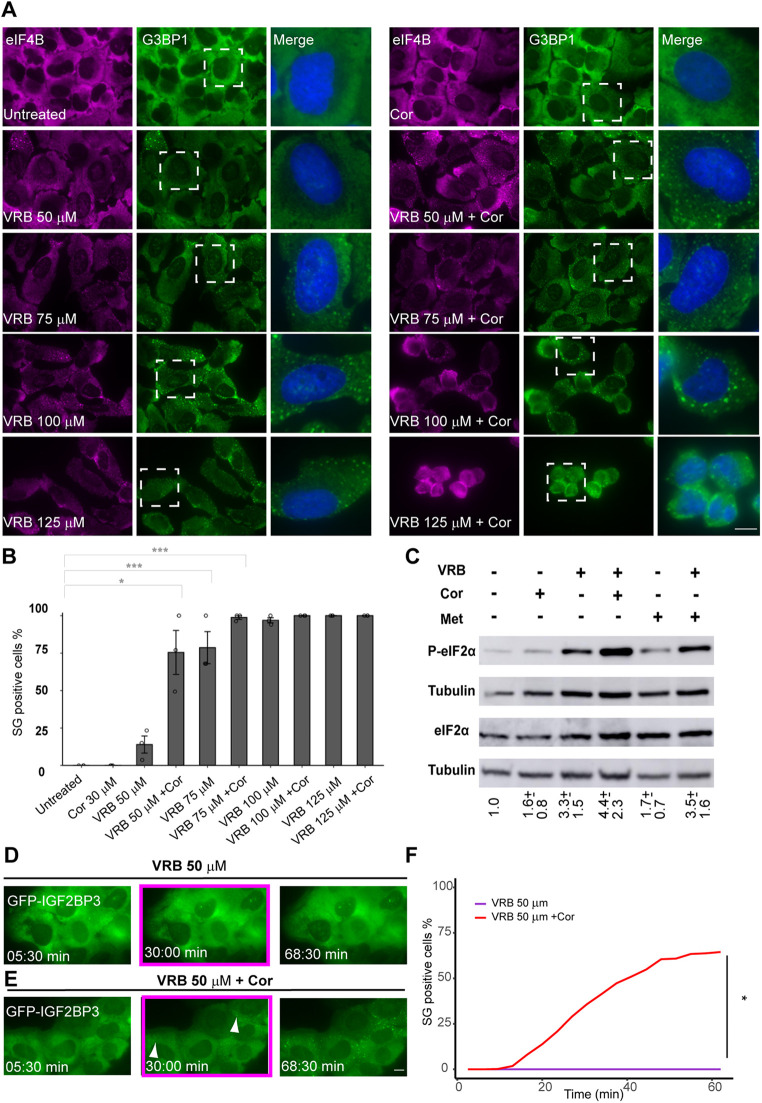
**Cortisone enhances the formation of VRB-induced SGs.** (A) The formation of SGs under VRB (50–125 µM) and cortisone (Cor; 300 µM) treatment for 1 h in U2OS cells was detected using anti-eIF4B (magenta) and anti-G3BP1 (green) SG markers. Hoechst 33342 DNA stain is shown in blue. Dashed boxes indicate regions shown in the enlarged merge images on the right. Scale bar: 10 µm. (B) Quantification of the population of SG-positive U2OS cells under the different treatment conditions as in A. Data are presented as mean±s.e.m., with each circle on the bar graph indicating a biological replicate (*n*=3). *P<0.05; ***P<0.001 [one-way ANOVA with Tukey's post hoc test for comparing treatments with non-constant values and two-tailed one-sample t-tests (against mean values of 0 or 100) for comparing treatments with constant values (either 0% or 100%) with treatments with non-constant values]. (C) VRB and cortisone increase phosphorylated eIF2α (P-eIF2α) levels. Western blot analysis of eIF2α and P-eIF2α levels in protein extracts from U2OS cells after addition of VRB (50 µM), Cor (300 µM) or methanol (Met, 3%), and their indicated combinations, for 1 h treatments. Tubulin was used as loading control. This experiment is representative of five separate repeats. Mean±s.d. fold change in P-eIF2α is designated under the lanes; the analysis was performed by normalizing the ratio of P-eIF2α to eIF2α for each treatment group to that of the untreated group. (D,E) Frames from time-lapse movies showing SG formation in U2OS cells stably expressing GFP–IGF2B under treatment with 50 µM VRB (D; see Movie 1) or with 50 µM VRB and 300 µM Cor (E; see Movie 2). Images were acquired every 3.5 min for ∼1 h (time shown as min:s). The boxed frames (∼30 min time points) show that no SGs were formed in the VRB-treated cells, whereas SGs were observed when cortisone was added alongside VRB (arrowheads, SGs). Scale bar: 10 μm. (F) Quantification of the population of SG-positive U2OS cells under the different treatment conditions as in D and E. Data were analyzed using a two-tailed one-sample *t*-test against a constant mean value of 0 to compare the fraction of SG-positive cells for the two treatments at the last time point (*P<0.05). Lines represent the mean of *n*=30 videos, with SG-positive cells quantified at 20 time points.

Next, cortisone was added to U2OS cells together with the VRB treatment, and a significant increase in the proportion of cells containing SGs was observed (Fig. 2A,B). Cortisone alone did not induce SG formation. The VRB concentration under which cortisone induces the most obvious effect in SG assembly was calibrated. A minority of cells in the population (15%) harbored SGs when treated with 50 µM VRB, whereas the addition of cortisone together with 50 µM VRB induced the formation of SGs in 75% of the cells. At higher concentrations of VRB (100 and 125 µM) the whole cell population contained SGs, and under these conditions, cortisone addition enhanced the retraction of the cytoplasm, implying increased cellular stress (Fig. 2A,B). Consequently, the 50 µM VRB conditions were found to be the preferred conditions to discern between the effects of VRB alone (low percentage of cells with SGs) and VRB together with cortisone (high percentage of cells with SGs). These conditions were further used to determine the role cortisone plays in SG assembly under VRB stress. To examine whether the SG assembly-enhancing effect of cortisone was the general outcome during microtubule disassembly, the related microtubule inhibitor VBL was tested, and the same effect of enhanced SG assembly in the cell population was observed (Fig. S2A). Indeed, the microtubule cytoskeleton was disassembled in VRB-treated as well as VRB- and cortisone-treated cells (Fig. S2B). The enhancement of SG formation by cortisone in the presence of VRB was seen in a variety of human and murine cell lines (Fig. S3). In mouse cells, cortisone alone had an effect on SG formation, as discussed below. Similar enhancement of SG formation in human and mouse cells was seen when another glucocorticoid, prednisone, was added to cells alongside VRB (Fig. S4).

### Cortisone augments the kinetics of SG formation under VRB stress conditions

It is well characterized that in mammalian cells, the assembly of SGs is initiated by phosphorylation of eIF2α (Kedersha et al., 1999). We examined the effects of VRB and cortisone on eIF2α phosphorylation. VRB alone increased the phosphorylation (∼3.3-fold) whereas cortisone only had a mild effect (Fig. 2C), in agreement with no inducing effect of cortisone on SG formation, as seen above (Fig. 2A,B). The phosphorylation increased (∼4.4-fold), compared to phosphorylation levels in untreated cells, when VRB and cortisone were administered together (Fig. 2C). Live-cell imaging of SG induction in U2OS cells stably expressing GFP–IGF2BP3 showed that the kinetics of SG assembly were enhanced under VRB and cortisone conditions. Specifically, SGs appeared by 30 min under combined VRB and cortisone treatment, whereas SGs were not observed at 30 min of VRB treatment (50 µM) or later (Fig. 2D–F; Movies 1,2).

**Fig. 3.**
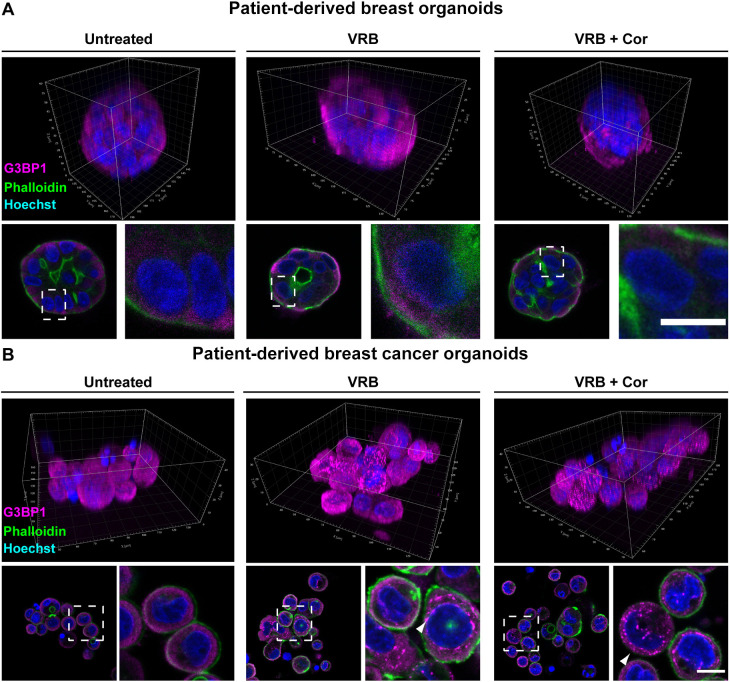
**VRB and cortisone induce the formation of SGs in patient-derived breast cancer organoids.** SG formation was examined when cortisone (Cor; 300 µM) was added with the VRB treatment (75 µM for 2 h) in patient-derived breast organoids originating from either (A) healthy tissue or from (B) cancerous tissue. SGs were detected using an anti-G3BP1 antibody (magenta). Phalloidin staining of actin (green) was used to detect the cell outline. Hoechst 33342 DNA stain is shown in blue. The top rows show 3D presentations of the organoids. Dashed boxes in the images of organoid sections indicate regions of cells shown in enlarged images on the right. Arrowheads mark SGs. Scale bars: 10 µm. Images are representative of three experiments.

We then tested the effect that cortisone had on SG formation in the breast-derived organoids from normal and cancer tissues. Strikingly, there continued to be no SG formation in the organoids derived from normal breast tissue even when cortisone was added with the VRB (Fig. 3A; Fig. S1). The formation of SGs upon VRB and cortisone treatment was observed in the patient-derived breast cancer organoids (Fig. 3B; Fig. S1). In summary, cortisone has an enhancing effect on SG assembly during VRB stress that is observed as an induction of the fraction of cells containing SGs, as an increase in the rate at which SGs form, and as a rise in the levels of eIF2α phosphorylation.

### Cortisone addition with VRB impairs the disassembly kinetics of SGs

Previous studies have demonstrated that polyadenylated [poly(A)+] mRNA is recruited to SGs under various stress conditions, suggesting that mRNAs transit in and out of SGs as a component of a modified 48S pre-initiation complex (Kedersha et al., 2002). In fact, the RNA component is crucial for the formation of RNA granules in cells by liquid–liquid phase separation (LLPS) (Van Treeck and Parker, 2018). Therefore, the RNA fraction in the SGs formed under cortisone conditions was stained. RNA fluorescence *in situ* hybridization (FISH) using a fluorescent oligo-dT probe to detect poly(A) tails showed a distinct poly(A)+ RNA population in the VRB- and VRB plus cortisone-treated U2OS cells (Fig. S5A). We also tested several endogenous RNAs (Fig. S5B–D) using single-molecule RNA FISH (smFISH): MKI67 mRNA, which encodes Ki67; the lncRNA NORAD; and IPO7 mRNA, which encodes importin 7 and which we had previously detected in SGs under arsenite stress conditions (Sheinberger et al., 2017). All were detected in SGs, specifically in the peripheral region of the SG, as has previously been documented for mRNA localization in SGs (Moon et al., 2019).

**Fig. 4.**
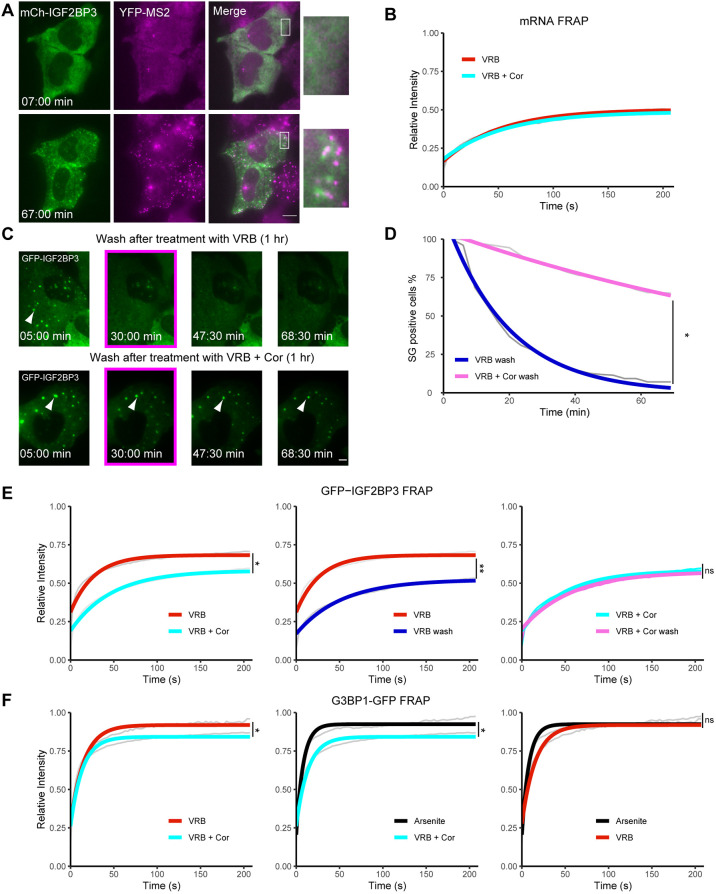
**Addition of cortisone to VRB modifies the formation and dissolving properties of SGs.** (A) Frames from Movie 3 showing the accumulation of β-actin mRNAs in SGs in U2OS cells at the indicated times (min:s) after VRB (75 µM) treatment. Images were acquired every 5 min for ∼1 h. The SGs are seen with mCherry–IGF2B (mCh–IGF2BP3; green) and the mRNAs with YFP–MS2-CP (magenta). Boxes indicate regions shown in magnified views on the right. Scale bar: 10 µm. Images are representative of four experiments. (B) FRAP recovery curves of YFP–MS2-CP-tagged β-actin mRNAs within SGs under VRB (75 µM; red curve) and VRB plus cortisone (VRB+Cor; 75 µM VRB, 300 µM cortisone; cyan curve) conditions for 1 h. Data are presented as an averaged data plot (gray line). Colored lines represent the best fit recovery curves for each treatment. (C) Frames for time-lapse movies (Movies 4 and 5) showing SG dissolution in U2OS cells stably expressing GFP–IGF2B. Cells were treated either with 75 µM VRB or with 75 µM VRB and 300 µM cortisone (VRB+Cor) for 1 h, and then washed with fresh medium. Images were acquired every 5 min for 1 h (time is shown as min:s). The boxed frames show that SGs (arrowheads) dissipated after ∼30 min following washout of VRB treatment but were still present more than 1 h after VRB+Cor treatment. Scale bar: 10 µm. Images are representative of four experiments. (D) Quantification of the population of SG-positive U2OS cells over time under the different treatment conditions as in C: wash after 1 h VRB treatment (75 µM; blue curve) and wash after 1 h VRB+Cor treatment (75 µM VRB, 300 µM cortisone; pink curve). Data represent the mean of 30 videos, with 20 time points used for quantification of SG-positive cells. Averaged data are plotted in gray, with colored lines showing best fit plots for SG-positive cells after rinsing off treatment. Data were analyzed using a two-tailed independent sample *t*-test at the last time point (*P<0.05). (E) FRAP recovery curves of GFP–IGF2BP3 in SGs. Left: VRB (75 µM; red curve) and VRB+Cor (75 µM VRB, 300 µM cortisone; cyan curve). Middle: VRB (red curve) and wash after VRB (blue curve). Right: VRB+Cor (cyan curve) and wash after VRB+Cor (pink curve). Treatments were for 1 h, and washout experiments were conducted within 30 min post washing. Data are presented as an averaged data plot (gray lines). Colored lines represent the best fit recovery curves for each treatment. (F) FRAP recovery curves of G3BP1–GFP in SGs. Left: VRB (75 µM; red curve) and VRB+Cor (75 µM VRB, 300 µM cortisone; cyan curve) for 1 h. Middle: arsenite (45 min, 0.5 mM; black curve) and VRB+ Cor (1 h; cyan curve). Right: arsenite (45 min, 0.5 mM; black curve) and VRB (1 h; red curve). For B,E,F, data are presented as an averaged data plot (gray line). Colored lines represent the best fit recovery curves for each treatment. Data were analyzed by one-way nested ANOVA. Post-hoc analysis was performed as pairwise comparisons defined by linear contrasts, and *P*-values were adjusted with the Benjamini–Hochberg (FDR) procedure. (*P<0.05; **P<0.01; ns, non-significant). Statistical information, including *n* values for all FRAP experiments, and details of curve fitting appear in the Materials and Methods section.

We next wanted to follow mRNA dynamics in SGs in living cells under these conditions. We used a U2OS cell clone containing a doxycycline-inducible β-actin (*ACTB*) gene under Tet-On control (Ben-Ari et al., 2010). The β-actin mRNA transcribed from this gene has a series of 24 MS2 sequence repeats in the 3′ UTR. These form a series of stem-loops that are subsequently bound by a fluorescent-tagged MS2 bacteriophage coat protein, YFP–MS2-CP, which can be used for tracking of mRNA in living cells (Bertrand et al., 1998). These mRNAs entered SGs under both VRB and VRB plus cortisone conditions. This recruitment was observed both in fixed (Fig. S5E) and living cells (VRB only; Fig. 4A; Movie 3)*.* To analyze the association dynamics of the β-actin mRNA with the SG structure, we used fluorescence recovery after photobleaching (FRAP). The mRNA signal in SGs exhibited a very slow recovery, with a substantial immobile fraction (∼50%) still remaining after 210 s of recovery (Fig. 4B). This indicates that a substantial portion of these mRNAs remained associated with the SGs for several minutes before being released back into the cytoplasm. Cortisone did not have an effect on the dynamics of β-actin mRNA association with the SGs.

We found that cortisone addition with VRB enhances the rates at which SGs assemble (Fig. 2D–F; Movies 1,2). Therefore, we used time-lapse imaging to test whether SG disassembly dynamics were modulated in the presence of cortisone. Indeed, most SGs dissolved rapidly in VRB-treated U2OS cells by ∼30 min after washout of VRB, whereas ∼70% of cells treated with VRB and cortisone remained positive for SGs even 1 h after washout (Fig. 4C; Movies 4,5). Quantifying this clearance in the cell population that was positive for SGs, we found that VRB-treated cells dropped from 100% positive for SGs to close to 0% after 1 h, while in cells treated with VRB and cortisone, SGs were maintained in ∼70% of cells (Fig. 4D). We therefore next examined whether the dynamic properties of proteins within the SGs had changed, thereby altering the dissolving properties of SGs. FRAP experiments were performed on two RBPs in SGs: IGF2BP3 and G3BP1. The results indicated that the addition of cortisone with VRB slowed the recovery dynamics of the SG proteins, compared to VRB treatment alone. These proteins exhibited a significantly larger immobile fraction when cortisone was added with the VRB compared to their immobile fractions in VRB-treated cells (Fig. 4E and F, left). Specifically, the immobile fraction of IGF2BP3 increased from 30% to 45%, while for G3BP1, the dynamics changed from a near complete recovery to a ∼20% fixed fraction. This means that the addition of cortisone increased the residence times of the proteins in the SGs, modifying their biophysical properties.

To further examine the impaired clearance of SGs in the presence of cortisone, FRAP was performed on IGF2BP3 within SGs in cells where the treatment had been rinsed away. We hypothesized that the SGs in VRB and cortisone-treated cells would have similar IGF2BP3 recovery curves in the presence of the treatment and after the treatment was rinsed away, because the SGs were still present after the cells were rinsed, and less dissolving of SGs took place. This is in contrast to SGs in the cells treated with VRB alone, which we expected to have a very different IGF2BP3 recovery curve after the VRB treatment was washed away. Due to the rapid dispersal of the SGs after VRB washout, we expected that they should recover less because few proteins would be re-entering the SGs. Indeed, we found that the IGF2BP3 recovery curve for VRB and cortisone-treated cells displayed a slight drop in overall recovery following washout of the treatment compared to that observed in the presence of the treatment; this drop in recovery was not statistically significant (Fig. 4E, right), due to minor dissolution of these SGs. In contrast, the VRB-treated cells exhibited a significant drop in IGF2BP3 recovery from ∼70% to only ∼50% after VRB washout (Fig. 4E, middle). We verified that the change in recovery of G3BP1 in the VRB plus cortisone conditions was not because more G3BP1 was found in the core of more SGs by treating cells with arsenite to cause a strong induction of SG formation. The FRAP recovery curves showed full recovery of G3BP1 under arsenite and VRB conditions (Fig. 4F, right), whereas full recovery of G3BP1 was not observed in VRB plus cortisone conditions (Fig. 4F, middle), implying that G3BP1 rapidly exchanges in most SGs, but in SGs induced by VRB plus cortisone treatment there was less exchange due to an alteration in the nature of the granules. In conclusion, cortisone affects the association dynamics of some of the core protein components with the SGs, leading to a less dynamic SG structure and impaired SG clearance.

### Cortisone treatment enhances cell death in VRB-treated cells

**Fig. 5.**
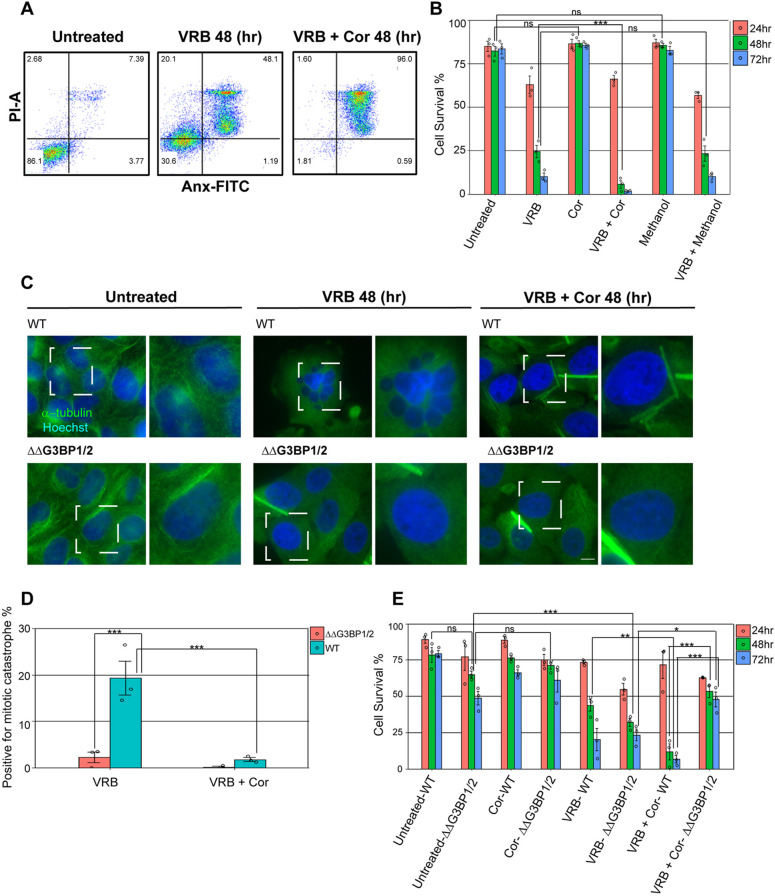
**Cortisone affects the viability of cells treated with VRB.** (A,B) Annexin V and PI analysis was performed on U2OS cells treated with VRB (10 µM), cortisone (Cor, 300 µM) and methanol (3%), individually and in combination, for 24, 48 and 72 h to monitor cell death under these conditions. (A) Two-dimensional dot plots showing annexin V (Anx–FITC) and PI (PI–A) signal in the different subpopulations detected by flow cytometry at 48 h. Numbers in each quadrant represent the percentage of cells in each of the populations detected. (B) The percentage of live cells (annexin V and PI-negative) under the different treatments at the indicated time points. Data are presented as mean±s.e.m., with each circle on the bar graph indicating a biological replicate (*n*=3). Data were analyzed by two-way ANOVA with Tukey's post hoc test (***P<0.001; ns, non-significant). (C) U2OS wild-type (WT) and ΔΔG3BP1/2 cells were treated with 10 µM VRB or with 10 µM VRB and 300 µM cortisone (VRB+Cor) for 48 h and stained to detect α-tubulin (green). Dashed boxes indicate regions shown in magnified views on the right. Mitotic catastrophe was observed by Hoechst 33342 DNA staining (blue) and can be seen in the boxed area in the VRB-treated WT cells. Scale bar: 10 μm. (D) Quantification of WT and ΔΔG3BP1/2 U2OS cells positive for mitotic catastrophe following treatment as described in C. Data are presented as mean±s.e.m., with each circle on the bar graph indicating a biological replicate [*n*=3; ***P<0.001; post-hoc analysis was performed as pairwise comparisons defined by linear contrasts, and *P*-values were adjusted with the Benjamini-Hochberg (FDR) procedure]. (E) Graphical representation of annexin V and PI analysis of WT and ΔΔG3BP1/2 U2OS cells, showing the percentage of live cells (annexin V and PI-negative). WT and ΔΔG3BP1/2 cells were treated with VRB (10 µM) and cortisone (300 µM), individually and in combination, for 24, 48 and 72 h. Data are presented as mean±s.e.m., with each circle on the bar graph indicating a biological replicate (*n*=3). Data were analyzed by two-way ANOVA with Tukey's post hoc test (*P<0.05; **P<0.01; ***P<0.001; ns, non-significant).

Since cancer patients are often treated simultaneously with chemotherapy and glucocorticoids (Herr et al., 2003), and because cortisone had a robust effect on SG formation, we examined whether the addition of cortisone would affect the viability of the cells treated with VRB. Viability assays were performed over several days using a lowered VRB concentration (10 µM). Examining cell viability using annexin V and propidium iodide (PI) labeling for the identification of apoptotic U2OS cells in a flow cytometry assay showed that VRB treatment gradually induced cell death, as expected (Fig. 5A,B; Fig. S6A). At 24 h, viability was reduced to ∼63%, and was prominently reduced from 48 h (∼24% viability), decreasing to ∼10% viability of the cell population at 72 h. However, the addition of cortisone with VRB dramatically reduced cell viability levels to ∼6% at 48 h, leading to almost complete cell death of the whole population at 72 h (∼1.5% viability). Methanol (the vehicle control) did not show any significant contribution to cell death, either alone or with VRB.

VRB is known to lead to a specific type of cell death called mitotic catastrophe, during which cells replicate their DNA without going through mitosis (Altinoz et al., 2018). Cells fail to maintain a mitotic arrested state over time (termed mitotic slippage), resulting in hyperploidization, which is a hallmark of cells in this condition, and finally they die. Indeed, a significant proportion of hyperploid cells was observed in the population of U2OS cells treated with VRB (Fig. 5C,D). Surprisingly, the addition of cortisone to VRB-treated cells led to a significantly reduced percentage of cells exhibiting mitotic catastrophe characteristics (Fig. 5D), indicating that cortisone abrogates the possibility of mitotic slippage, leading to an enhanced effect on cell death.

In light of these results and taking into consideration the finding that cortisone impairs the dissolving properties of SGs (Fig. 4C–F), we sought to determine whether viability levels could be linked to SG components. It was previously shown that U2OS cells lacking the SG nucleating proteins G3BP1 and G3BP2 (ΔΔG3BP1/2 cells) did not form SGs under certain conditions such as chronic starvation and VRB treatment (Kedersha et al., 2016; Reineke et al., 2018). Annexin V and PI analysis was performed on ΔΔG3BP1/2 and wild-type U2OS cells. VRB treatment gradually increased cell death in both ΔΔG3BP1/2 and wild-type U2OS cells, as expected (Fig. 5E; Fig. S6B). Importantly, the addition of cortisone to ΔΔG3BP1/2 cells alongside VRB treatment revealed an increase in cell survival at 48 and 72 h compared to that of ΔΔG3BP1/2 cells treated with VRB alone. Specifically, cell viability of ΔΔG3BP1/2 cells treated with VRB alone after 48 and 72 h was 32% and 23%, respectively, while the combination of VRB and cortisone led to relatively increased ΔΔG3BP1/2 cell survival after 48 and 72 h (53% and 48%, respectively). Moreover, the cell viability of ΔΔG3BP1/2 cells treated with VRB and cortisone for 48 and 72 h was higher than that of wild-type U2OS cells in the same conditions, and ΔΔG3BP1/2 cells showed a negligible population of mitotic catastrophes under VRB treatment for 48 h compared to the wild-type cells (∼2.5% and ∼19%, respectively; Fig. 5C,D). When the ΔΔG3BP1/2 cells were rescued by the expression of G3BP1 (Marmor-Kollet et al., 2020), a similar percentage of cells positive for mitotic catastrophe was observed as for the U2OS wild-type cells (Fig. S7A,B). In the cell viability analysis, there was a significant difference between ΔΔG3BP1/2 cells and the rescued cells under VRB plus cortisone conditions. The addition of cortisone to the rescued cells did not trigger cell survival as was seen in the ΔΔG3BP1/2 cells (Fig. S7C). Taken together, the data suggest that these SG components play a role in cell death and might be important in the mitotic catastrophe pathway during VRB-mediated cell death. The increase in cell viability induced by cortisone in G3BP1/2-depleted cells, together with the lack of SGs, implies that cortisone promotes cell death when given with VRB.

### Cortisone can induce the formation of SGs in mouse cells

**Fig. 6.**
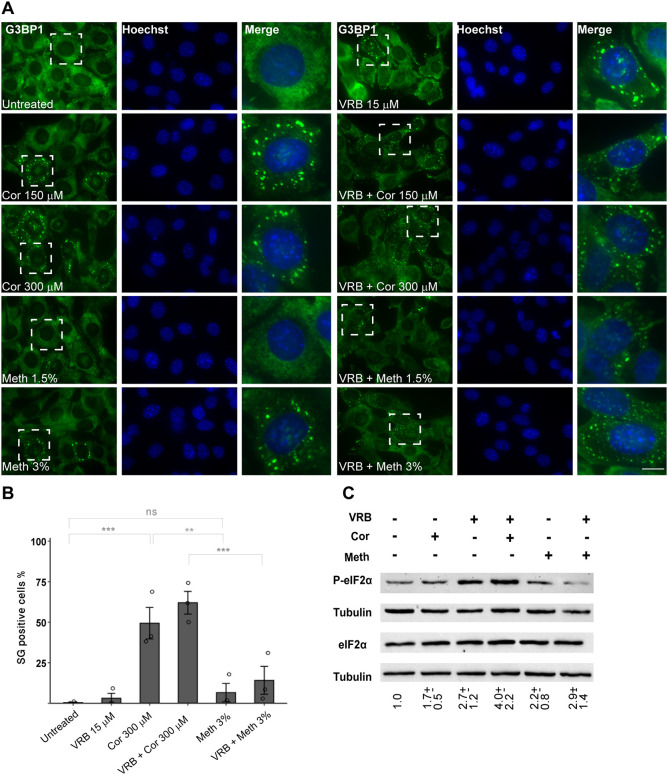
**Cortisone induces the formation of SGs in MEFs.** (A) The formation of SGs was detected in MEFs under the following treatments for 1 h: cortisone (Cor, 150 µM or 300 µM), methanol (Meth, 1.5% or 3%), VRB (15 µM) and their combinations, as indicated. SGs were stained for G3BP1 (green). Hoechst 33342 DNA stain is shown in blue. Dashed boxes indicate regions shown in enlarged images on the right. Scale bar: 10 µm. (B) The percentage of SG-positive cells in the MEF population was measured. Bar graph illustrates the mean±s.e.m., with each circle on the bar graph indicating a biological replicate (*n*=3). Data were analyzed using a one-way ANOVA with Tukey's post hoc test (**P<0.01; ***P<0.001; n.s., non-significant). (C) VRB and cortisone increase phosphorylated eIF2α (P-eIF2α) levels in MEFs. Western blot analysis of eIF2α and P-eIF2α protein levels in protein extracts from MEFs after treatment with VRB (15 µM), cortisone (Cor, 150 µM) or methanol (Meth, 3%), and their combinations, for 1 h. Tubulin is shown as a loading control. This experiment is representative of three separate repeats. Mean±s.d. fold change in P-eIF2α is designated under the lanes; the analysis was performed by normalizing the ratio of P-eIF2α to eIF2α for each treatment group to that of the untreated group.

We found that cortisone alone had an SG-inducing effect in mouse cells, namely, mouse embryonic fibroblasts (MEFs) (Sullivan et al., 1999) and HT-22 mouse hippocampal neuronal cells (Fig. S3D,E). We tested this in other immortalized MEFs (Lionnet et al., 2011) and saw the same phenomenon. MEFs showed higher sensitivity to the VRB treatment, and so lower concentrations of VRB were used in comparison to those used in experiments with human cells. VRB at 15 µM had a minor effect on SG assembly in these cells, whereas the coupled administration of VRB and cortisone (300 µM) for 1 h led to an increase in SG formation in ∼65% of the cell population (Fig. 6A,B). Cortisone on its own (at 300 µM) induced the formation of SGs in ∼50% of the cells. Similar results were obtained when cortisone was combined with VRB. Since cortisone is dissolved in methanol and these cells are sensitive, we examined MEFs treated with methanol alone. SGs formed in only a small fraction of the cell population (3%), which was significantly different from the SG formation following cortisone treatment (Fig. 6B), and therefore we conclude that the increase in SGs is due to cortisone addition.

Finally, in MEFs, as in the human cell lines, eIF2α phosphorylation was induced by VRB alone (∼2.7-fold) and by cortisone alone (∼1.7-fold), which correlated with the observed induction in the assembly of SGs by VRB or by cortisone alone in these cells (Fig. 6C). Addition of cortisone had an additive effect on the VRB-induced phosphorylation of eIF2α in MEFs (∼4-fold), as was observed in U2OS cells (Figs 6C and 2C). Altogether, we found that cortisone induces the formation of SGs in mouse and human cells and increases the phosphorylation of eIF2α over and above VRB treatment alone.

### VRB and cortisone affect eIF2α phosphorylation by two different pathways

**Fig. 7.**
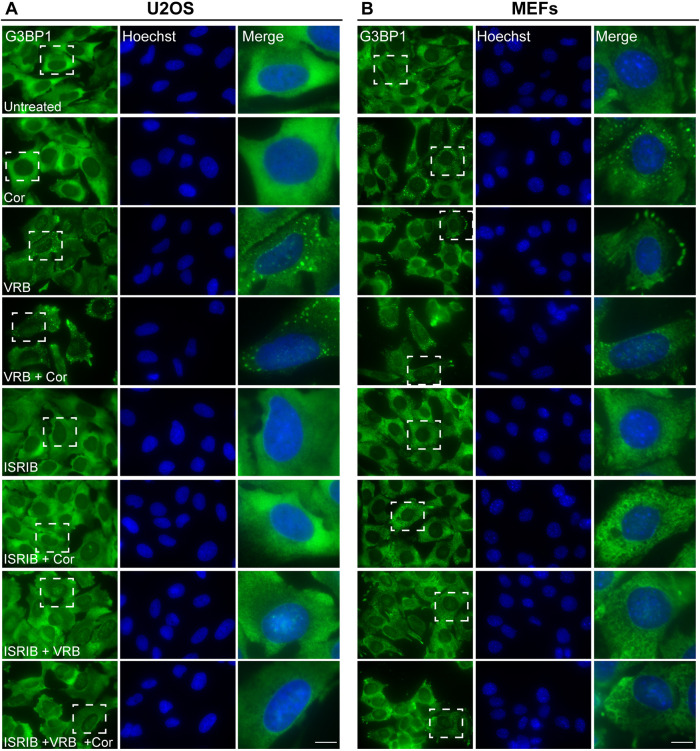
**ISRIB blocks SG formation induced by VRB and cortisone.** (A) U2OS cells and (B) MEFs treated with ISRIB (5 µM) for 2 h before addition of VRB (75 µM for U2OS cells, 15 µM for MEFs), cortisone (Cor, 300 µM) or both for 1 h, as indicated. SGs were detected using G3BP1 (green) as an SG marker. Hoechst 33342 DNA stain is shown in blue. Dashed boxes indicate the regions shown in enlarged images on the right. Scale bars: 10 µm. Images shown are representative of three experiments.

Phosphorylation of the translation initiation factor eIF2α is the outcome of the ISR (Wek et al., 2006). Recently, a specific small molecule inhibitor of the ISR was identified, termed Integrated Stress Response Inhibitor (ISRIB) (Sidrauski et al., 2013; Tauber and Parker, 2019). Surprisingly, ISRIB does not decrease the phosphorylation of eIF2α, rather it reverses effects that occur downstream of the phosphorylation (Sidrauski et al., 2015). To examine whether VRB and cortisone can elicit their effects through the ISR and induce SG assembly through eIF2α phosphorylation, we treated cells with ISRIB together with the VRB and cortisone treatments. Indeed, ISRIB inhibited SG formation under VRB and VRB plus cortisone treatments in U2OS cells and MEFs (Fig. 7A,B). ISRIB also blocked the effect of cortisone alone in inducing SG assembly in MEFs (Fig. 7B).

**Fig. 8.**
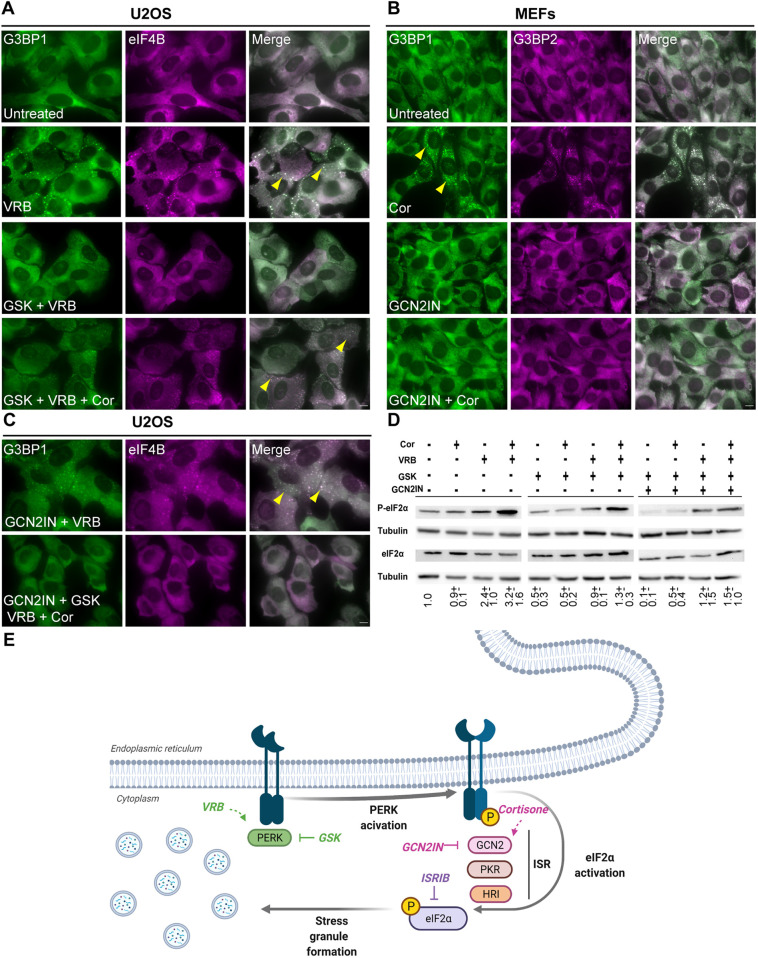
**GSK blocks SG formation induced by VRB, whereas GCN2 inhibitor blocks cortisone-induced SGs in MEFs.** (A) U2OS cells treated with the PERK inhibitor GSK (40 µM) for 2 h before addition of VRB (75 µM) or VRB (75 µM) and cortisone (Cor, 300 µM) for 1 h, as indicated. SGs are stained with antibodies to detect G3BP1 (green) and eIF4B (magenta). (B) MEFs treated with a GCN2 inhibitor (GCN2IN, 4 µM) for 2 h before Cor (300 µM) addition, as indicated. SGs are labeled with antibodies to detect G3BP1 (green) and G3BP2 (magenta). (C) U2OS cells treated with GCN2 inhibitor (4 µM) and GSK (40 µM) for 2 h before addition of VRB (75 µM) and Cor (300 µM) for 1 h, as indicated. SGs are labeled for G3BP1 (green) and eIF4B (magenta). In A–C, arrowheads indicate SGs. Scale bars: 10 µm. Images are representative of three experiments. (D) Western blot analysis of eIF2α and phosphorylated eIF2α (P-eIF2α) protein levels in U2OS cells after treatments with GCN2 inhibitor (4 µM) and GSK (40 µM) for 2 h before VRB (75 µM) and Cor (300 µM) addition for 1 h, as indicated. Tubulin was used as a loading control. This experiment is representative of three separate repeats. Mean±s.d. fold change is designated under the lanes; the analysis was performed by normalizing the ratio of P-eIF2α to eIF2α for each treatment group to that of the untreated group. (E) Scheme depicting the ISR pathway and SG formation by VRB and cortisone.

We then used specific inhibitors to determine through which pathways the VRB and cortisone act. GSK2656157 (PERK inhibitor II; referred to hereafter as GSK) is a specific inhibitor of PERK (Axten et al., 2012). GSK treatment (Axten et al., 2013) inhibited the formation of SGs in U2OS cells during VRB treatment but did not block SG formation when cortisone was added alongside VRB (Fig. 8A). To dissect the pathway cortisone works through we used the MEF cell line, since in these cells, cortisone alone induced SG formation (Fig. 6A). The GCN2 kinase was inhibited in MEFs using a specific inhibitor, and under these conditions, cortisone-induced SG formation was blocked (Fig. 8B). In U2OS cells, many cells were positive for SGs when the GCN2 inhibitor was administered prior to VRB (Fig. 8C), implying that VRB is not involved in the GCN2 kinase pathway. When both pathways were blocked, no SGs formed (Fig. 8C).

The phosphorylation of eIF2α was then examined. Combined treatment with VRB and cortisone increased the levels of phosphorylated eIF2α compared to those observed upon VRB treatment alone (Fig. 8D). When PERK was inhibited using GSK, there was a reduction in eIF2α phosphorylation in VRB-treated cells, but this decrease was not seen in the cells treated with both VRB and cortisone. This correlates with the immunofluorescence results showing that SG formation continued when cortisone was added alongside VRB in the presence of GSK (Fig. 8A). When GCN2 and PERK were inhibited together there was a reduction in eIF2α phosphorylation levels in both the VRB- and the VRB plus cortisone-treated cells (Fig. 8D). This too correlated with the immunofluorescence results and the observed reduction in SG formation (Fig. 8C). We conclude that VRB and cortisone activate two different arms of the ISR – PERK is activated in response to VRB, whereas GCN2 is activated in response to cortisone (Fig. 8E) – and this can explain the additive effects of cortisone with respect to SG dynamics and cell viability.

## DISCUSSION

In this study we examined the effect of glucocorticoids, specifically cortisone, on vinca alkaloid-induced SG formation and found that SG assembly and disassembly dynamics, the signal transduction pathways of the ISR, cell viability, and cell death pathways were all influenced by the addition of cortisone. Not much is known about the effects of hormones on the induction of SGs in cells. It has been shown that three non-glucocorticoid hormones, β-estradiol, progesterone and stanolone, prevent the creation of arsenite-induced SGs in HeLa cells (Timalsina et al., 2018). In contrast, these hormones do not prevent SG formation in other cancerous cell lines, such as HCT116, MCF7, MDA-MB-468, PANC-1 and OVCAR-5 (Timalsina et al., 2018), suggesting that hormone-mediated SG formation is cell-type specific. Some studies suggest that glucocorticoids can cause oxidative stress via reactive oxygen species (Almeida et al., 2011; Yokoyama et al., 2017). In this study, differences between mouse and human cell lines were identified. Cortisone on its own induced SG formation in mouse cells, whereas in human cell lines the addition of cortisone to VRB-treated cells increased the frequency of SG-positive cells. Similar results were obtained with the glucocorticoid prednisone.

Stress-induced phosphorylation of eIF2α is typically connected to SG formation, and this pathway was activated by cortisone and VRB in human and mouse cells. eIF2α phosphorylation on serine 51 results in severe decline in *de novo* protein synthesis and is an important strategy in the cell's armory against stressful insults. VRB promotes SG formation via both eIF2α phosphorylation by PERK and the interference of eIF4F complex assembly (Szaflarski et al., 2016). Here, we investigated the molecular mechanism of SG formation triggered by VRB and cortisone administration. VRB alone upregulated levels of phosphorylated eIF2α, both in U2OS cells and MEFs, as expected (Szaflarski et al., 2016). Cortisone alone upregulated levels of phosphorylated eIF2α and SG formation in MEFs. The highest eIF2α phosphorylation levels and SG formation in U2OS cells and MEFs were observed when VRB and cortisone were administrated together. Both VRB and cortisone activate the ISR and trigger eIF2α phosphorylation, since ISRIB, which inhibits the ISR downstream of the phosphorylation (Sidrauski et al., 2013, 2015), inhibited VRB-induced, cortisone-induced and VRB and cortisone-induced SG formation. Specifically, VRB activated the PERK pathway (Szaflarski et al., 2016), as shown by experiments using the inhibitor GSK, while addition of cortisone overcame this inhibition. To pinpoint the kinase triggered by cortisone, we tested MEFs in which cortisone alone induced SG formation. When using an inhibitor of the GCN2 kinase, cortisone-induced SGs did not form. Returning to human cells, the combined use of GSK and the GCN2 inhibitor blocked SG formation when VRB and cortisone were administered together. We note that methanol, in which cortisone is solubilized, also had a minor inducing effect on eIF2α phosphorylation. However, in the viability assays methanol had no effect, and the induced death was attributed to the cortisone. Also, with respect to SG formation, methanol had no significant inducing effect in the MEFs, and for both U2OS cells and MEFs, cortisone always drove SG formation to the highest levels when added alongside VRB. Altogether, as VRB activates PERK and cortisone activates GCN2, the combined response is stronger, suggesting a shift from a sub-lethal to a more lethal stress. High levels of phosphorylated eIF2α are known to induce apoptosis and pro-apoptotic factors, including CHOP among others (Lasfargues et al., 2012). Enhanced SG formation is a consequence of eIF2α phosphorylation and a marker of the enhanced cell reaction to stress.

SGs can impact cell death and survival (Park et al., 2020). Many studies have demonstrated the connection between chemotherapeutic resistance and SG formation in cancer cells (Anderson et al., 2015; Arimoto et al., 2008; Fournier et al., 2010; Gao et al., 2019; Kaehler et al., 2014; Mason et al., 2011; Woldemichael et al., 2012). For instance, the appearance of SGs in HeLa cells after exposure to the proteasome inhibitor bortezomib, which is used for the treatment of multiple myeloma, and the subsequent resistance of cells to bortezomib-induced apoptosis, have indicated that SG assembly might act as a cellular mechanism of resistance to chemotherapy (Arimoto et al., 2008; Fournier et al., 2010; Gareau et al., 2011). This protective effect is reversed when eIF2α phosphorylation is inhibited, just as inhibition of PKR is associated with increased tumor chemosensitivity (Mokas et al., 2009; Pataer et al., 2009). In another study, the anti-metabolite 5-fluorouracil (5-FU) – which is used in the treatment of head, neck, breast and colorectal cancers – has been found to also induce the assembly of SGs by the phosphorylation of eIF2α (Kaehler et al., 2014). The assembly of SGs has been associated with enhanced metastasis (Somasekharan et al., 2015). G3BP1-deficient tumors in mouse models have fewer SGs and are less prone to metastasis (Somasekharan et al., 2015).

VRB-induced SGs have been shown to promote cell survival and therefore decrease treatment efficiency (Szaflarski et al., 2016). This emphasizes the importance of finding a substance that will interfere with the cellular resistance triggered by the administration of VRB alone. We showed that the addition of cortisone alongside VRB in U2OS cells promoted cell death, predominantly after 48 h. It has previously been shown that a cotreatment of chemotherapy and glucocorticoids can affect cell death. For instance, glucocorticoid addition induces apoptosis in malignant lymphoid cells (Kofler, 2000; Planey and Litwack, 2000); however, it has an anti-apoptotic effect on cervical carcinoma cells (Herr et al., 2003). To determine whether the decrease in cell viability is due to the effect that cortisone has on SG formation, ΔΔG3BP1/2 cells, which do not form SGs under VRB conditions (Szaflarski et al., 2016), were treated with VRB and cortisone. In contrast to the effects on wild-type U2OS cells, the addition of cortisone led to an increase in cell viability in VRB-treated ΔΔG3BP1/2 cells, indicating that cortisone induces the formation of SGs linked to cell death. It has previously been shown that there are certain stress types, such as selenite stress and chronic starvation, that induce SGs linked to cell death. Under those stress conditions, ΔΔG3BP1/2 cells have been shown to have higher cell viability compared to that of wild-type cells (Fujimura et al., 2012; Reineke et al., 2018). However, it is important to mention that, in addition to its role in SGs, G3BP1 is also involved in various mechanisms that are linked to cancer and cell viability (Zhang et al., 2019). Therefore, we cannot conclude that the effect of G3BP1 on cell death is limited to the absence of SGs in the ΔΔG3BP1/2 cells.

It is known that VRB leads to mitotic catastrophe (Altinoz et al., 2018). The addition of cortisone to VRB-treated U2OS cells as well as the lack in SG formation in G3BP1/2-depleted cells prevented cells from ending up in a mitotic catastrophe. Rescuing the cells by the reintroduction of G3BP1 reversed these phenotypes, increasing mitotic catastrophe and reducing cell viability. This suggests that both SG assembly and the addition of cortisone play a role in the cell death pathways. Since mitotic catastrophe was observed in the cells treated with VRB after 48–72 h, but not after 24 h, we suggest that the possibility to undergo mitotic catastrophe is determined by cell death kinetics. In normal cells, the addition of cortisone to VRB leads to a decrease in cell survival; therefore, cell death is encouraged and a negligible fraction of cells that are positive for mitotic catastrophe (compared to cells treated with VRB alone) are observed. This is also compatible with the findings that there is a positive correlation between prolonged mitotic arrest and cells resistance to apoptosis (Chumduri et al., 2015). Although VRB-induced SGs promote cell survival (Szaflarski et al., 2016), the lack of SGs in ΔΔG3BP1/2 cells increases their propensity for cell death, hence, only a minor fraction of the cells is observed to be positive for mitotic catastrophe.

The data we obtained suggest that the addition of cortisone alongside VRB alters SG characteristics, linking them to cell death rather than cell survival. SGs are dynamic membraneless granules that form by LLPS (Aguzzi and Altmeyer, 2016; Molliex et al., 2015) and have the physical properties of a viscous liquid droplet (Bracha et al., 2019; Hyman et al., 2014). It is suggested that SGs play a role in the progression of neurological diseases, since SG proteins are found in amyotrophic lateral sclerosis (ALS)-associated aggregates that seem to relate to SGs (Adjibade et al., 2015; Anderson et al., 2015; Marmor-Kollet et al., 2020; Siwach and Kaganovich, 2017; Wolozin and Ivanov, 2019). The physical properties of these aberrant SGs were found to be modified, with some of the proteins exhibiting reduced recovery rates in dynamics experiments and forming gel-like or even solid SGs (Mateju et al., 2017; Weisberg et al., 2012). The accumulation of such harmful aggregates over time in cells that cannot repair the damage is suggested to be an avenue for driving the neurodegeneration process. Photobleaching experiments have shown that, like many other membraneless organelles and granules in cells, the protein and RNA components of these structures are continuously shuttling in and out. SGs and PBs exchange both RNA and protein molecules. The exchange rate for proteins ranges from seconds to minutes, while for mRNA, the dynamics of exchange are less rapid and occur in the range of minutes. For instance, it has been shown that some endogenous mRNAs in SGs exchange slowly, with residence times of several minutes, while some mRNAs remain immobile (Aizer et al., 2014; Andrei et al., 2005; Bley et al., 2015; Kedersha et al., 2005; Mollet et al., 2008; Niewidok et al., 2018; Sheinberger and Shav-Tal, 2017; Stohr et al., 2006; Zhang et al., 2011).

Changes in the dynamic properties of SG formation and disassembly were observed when cortisone was added to the VRB treatment. SGs assembled more rapidly but dissolved more slowly. Looking specifically at an mRNA and at some protein components of SGs using FRAP showed that cells that received cortisone in addition to VRB showed higher immobile fractions of G3BP1 and IG2BP3 proteins in the SG structures compared to those of cells treated with VRB alone. Dynamics of the RNA fraction tested by FRAP did not change in response to cortisone, and the exchange dynamics with the cytoplasm remained very slow. Considering the heterogeneity of mRNA content within SGs and the different subtypes of SGs identified thus far (Advani and Ivanov, 2019), it is possible that mRNA immobility in the SGs would increase even more in response to cortisone, thus contributing to the low clearance of the SGs. As for the proteins tested, rinsing of the cells to remove the stressors demonstrated the persistence of the SG structures formed in the presence of cortisone. This implies that the biophysical properties of the SGs were impaired, and that the SGs were less capable of dissolving even though the stressor was not present. The continued presence of SGs may be linked to cell death, as they cannot be efficiently cleared by the cells, similar to the situation seen during neurodegeneration.

Most SG-related research has been conducted on the cellular level, and it is of interest to understand whether the rules of SG formation also follow in tissues. VRB is given as treatment for metastatic breast cancer (Aapro and Finek, 2012); hence, we examined the effect of treatments with VRB and cortisone on organoids derived from healthy breast tissue and metastatic breast cancer. We found that the effects of the VRB chemotherapy on these cell types were quite different. Specifically, the formation of SGs was induced only in the cancer cell organoids and not in the organoids originating from healthy tissues. Further examination of organoids from different origins is required, because the differences observed could be due to tissue origins rather than from them being from healthy or cancerous tissues. At present, SGs have been detected in iPSC-derived cerebral organoids. A study on neurodegeneration has found that mutant tau mRNA and protein interact with the ELAVL4 protein in neurons to promote the expression of SG proteins TIA-1 and G3BP1, including colocalization in SGs, contributing to impaired function of glutamatergic neurons (Bowles et al., 2021). Another study has generated cerebral organoids using iPSCs derived from healthy individuals and compared these to organoids generated using iPSCs derived from patients with Alzheimer's disease (AD), finding an acceleration in SG formation in the AD patient-derived organoids, especially when the strongest genetic risk factor for AD, the *APOE4* allele, was present (Zhao et al., 2020). Taken together, these studies suggest that SGs do not tend to form in healthy tissues under VRB treatment, but their assembly can be induced under diseased states. Further study on organoids from cancer origins should show how they can serve as important cell systems for the examination of the therapeutic potential of new medications.

## MATERIALS AND METHODS

### Plasmid construction

The GFP–IGF2BP3 plasmid was received from Stefan Hüttelmaier (Martin Luther University, Halle–Wittenberg, Germany; Stohr et al., 2006). For mCherry–IGF2BP3 construction, both GFP–IGF2BP3 and mCherry-C1 (a gift from Robert Singer, Albert Einstein College of Medicine, NY) plasmids were cut with AgeI and EcoRI, and after extracting the products from a 1% agarose gel, the mCherry insert was ligated into the IGF2BP3 vector. Then, an adaptor was used to insert a single nucleotide to correct a frameshift in the ORF of the IGF2BP3. G3BP1–GFP under Tet-On control was obtained from Eran Hornstein (Weizmann Institute of Science, Rehovot, Israel; Marmor-Kollet et al., 2020).

### Cell culture and transfections

Human U2OS cells (from the ATCC) were maintained in low glucose DMEM (Biological Industries, Beit-Haemek, Israel) supplemented with 10% fetal bovine serum (FBS; HyClone Laboratories, Logan, UT). HeLa (from the ATCC), A549 (gift from Amit Tzur, Bar-Ilan University, Israel), MCF7 and HT-22 (both gifts from Uri Nir, Bar-Ilan University, Israel) and MEFs (Lionnet et al., 2011; Sullivan et al., 1999) were maintained in high glucose Dulbecco's modified Eagle's medium (Gibco, USA) containing 10% FBS. U2OS cells containing the inducible β-actin gene (Ben-Ari et al., 2010) were induced using doxycycline (Sigma; 15 µg/ml) overnight. ΔΔG3BP1/2 cells were obtained from Nancy Kedersha and Pavel Ivanov (Brigham and Women's Hospital and Harvard Medical School, Boston, MA; Kedersha et al., 2016). G3BP1 rescued cells were obtained from Eran Hornstein (Marmor-Kollet et al., 2020). U2OS cells underwent cell line authentication. Cells were regularly tested for contamination using fluorescence microscopy and PCR.

Arsenite (0.25 mM or 0.5 mM; Sigma) was added to the medium for up to 45 min. VRB (10–125 µM; Sigma; concentration based on Szaflarski et al., 2016) and VBL (50–75 µM; Sigma) were added for different times, as designated. Cortisone (150–300 µM; Sigma), prednisone (300 µM; Sigma), methanol (1.5–3%; Daujung Siheung-si, Korea) were added as designated. For ISR treatments, the following compounds were used: ISRIB (5 µM; Sigma), GSK2656157 (40 µM; EMD Millipore) and GCN2 inhibitor (4 µM; MedChem Express).

For transfections, cells were transfected with 1–5 μg of plasmid DNA and 40 μg of sheared salmon sperm DNA (Sigma) using electroporation (Gene Pulser Xcell, Bio-Rad). Stable expression of GFP–IGF2BP3 was obtained by cotransfection with GFP–IGF2BP3 (10 µg) and puromycin resistance (300 ng) plasmids using electroporation and subsequent selection with puromycin (1 μg/ml; Invivogen, San Diego, CA).

### Organoids

For the establishment of patient-derived breast organoids, normal breast tissue was obtained via the Sheba Tissue Bank (Chaim Sheba Medical Center, Ramat Gan. Israel), organoids are established and maintained according to a published protocol (Dekkers et al., 2020). Briefly, BR33N organoids are derived from normal breast tissue resected during lumpectomy due to T1 hormone receptor positive breast cancer of a 85-year-old woman. No neoadjuvant treatment was administered prior to surgery. For organoid establishment, fresh tissue was mechanically and enzymatically digested, isolated cells were plated in adherent Cultrex growth-factor-reduced basement membrane extract (BME) type 2 drops and overlaid with optimized organoid culture medium (Dekkers et al., 2020) containing 10% R-spondin-1-conditioned medium (RCM) produced from HEK293 HA–Rspo1–Fc cells (Cultrex® HA–R-spondin-1–Fc 293T cells; 3710-001-01), 20% Wnt3a-conditioned medium (WCM) produced from L cells stably transfected with pcDNA3.1-Zeo-mouse Wnt3a, and 10% Noggin-conditioned medium (NCM) produced from HEK293 cells stably transfected with pcDNA3-mouse NEO insert (to confer neomycin resistance; cells for WCM and NCM production were kindly provided by the Hubrecht Institute, Utrecht, The Netherlands). Patient-derived breast cancer organoids were acquired from Hubrecht Organoid Technology (HUB, Utrecht, The Netherlands). HUB-01-C2-152 organoids are derived from an ER−, PR−, HER2+ breast cancer metastasis into a muscle of a 47-year-old woman. The patient was treated with tamoxifen, letrozole and olaparib before the tissue was obtained. Thawed organoids were plated in BME and covered with optimized growth medium (Sachs et al., 2018) containing 10% RCM and 10% NCM. Medium was changed every 4 days, and organoids were passaged every 2 weeks using mechanical shearing with (normal breast organoids) or without (breast cancer organoids) using TrypLE Express (Invitrogen, 12605036). Use of human tissues via Sheba Tissue Bank was approved by the local ethics committee and by the Associate Director at the Sheba Medical Center (approval no. 7188-20-smc), and informed consent was obtained for all tissue donors. Investigations were conducted according to the principles expressed in the Declaration of Helsinki.

For immunostaining, whole organoids were suspended in BME, plated in a µ-Slide 18 Well (81816, ibidi) and covered with appropriate growth medium overnight or longer. Following treatments, the organoids were fixed in 4% paraformaldehyde (PFA) for 30 min, permeabilized with 0.3% Triton X-100 in phosphate-buffered saline (PBS) for 30 min, then blocked with 3% BSA in PBST (0.01% Triton X-100 in PBS) for 1 h. The organoids were incubated with a primary antibody (mouse anti-G3BP1; 1:200; Abcam, ab56574) for 2 h at room temperature (RT), then for 1 h at RT with a secondary antibody (Alexa Fluor 647-labeled donkey anti-mouse IgG; Life Technology) together with phalloidin–FITC (5 µM; Sigma). Antibodies were diluted in 3% BSA in PBST, and incubations were followed by three washes of 5 min with PBST. DNA staining was performed with Hoechst 33342 for 10 min, and organoids were then covered with PBS.

### Western blotting

Cells were washed in cold PBS, and proteins were extracted using immunoprecipitation (IP) lysis buffer (ThermoFisher Scientific) containing 10 mM Na fluoride (Sigma), 10 mM Na orthovanadate (Sigma), 1 mM protease inhibitor cocktail (Sigma) and 1 mM PMSF (Sigma). The samples were then placed on ice for 20–25 min. The resulting lysate was centrifuged at 14,000 rpm (20,817 ***g***) for 10 min at 4°C. Then, 20–40 µg/µl of protein per lane was run on SDS-polyacrylamide gels and transferred to a nitrocellulose membrane (0.45 µm; Bio-Rad). The membrane was blocked in 5% BSA for 1 h at RT or overnight at 4°C, and then probed with a primary antibody for 2 h at RT followed by incubation with an HRP-conjugated goat anti-rabbit IgG secondary antibody (Abcam, ab7090) for 1 h at RT. For loading control, the membranes were reprobed with an anti-α-tubulin antibody. Immunoreactive bands were detected using an enhanced chemiluminescence kit (ECL, Pierce). Primary antibodies used were rabbit anti-eIF2α (Cell Signaling Technology, 9722; 1:1000), rabbit anti-phospho-eIF2α (Abcam, ab32157; 1:1000) and rabbit anti-α-tubulin (Abcam, ab4074; 1:000). Experiments were performed at least three times. Original blots can be seen in Fig. S8. To normalize the different bands to control conditions and obtain a relative value, the ratio of phosphorylated eIF2α to eIF2α was calculated in the untreated group. Each treatment group was normalized to this value by taking the ratio of phosphorylated eIF2α to eIF2α and dividing it by the normalizing value from the control group. The standard deviation was also calculated. Quantification of band intensity from the blots was done using ImageJ software (NIH, Bethesda, MD).

### Immunofluorescence

Cells were grown on coverslips, washed with PBS and fixed for 20 min in 4% PFA. Cells were then permeabilized in 0.5% Triton X-100 for 3 min. After blocking, cells were immunostained for 1 h with a primary antibody, and after subsequent washes the cells were incubated for 1 h with fluorescently labeled secondary antibodies. Primary antibodies: goat anti-TIA-1 (Abcam, ab40693; 1:200), rabbit anti-eIF4B (Abcam, ab68474; 1:200), mouse anti-G3BP1 (Abcam, ab56574; 1:200), rabbit anti-G3BP2 (Abcam, ab86135; 1:1000), rabbit anti-α-tubulin (Abcam, ab4074; 1:400). Secondary antibodies: Alexa Fluor 488-labeled goat anti-mouse IgG and anti-rabbit IgG, Alexa Fluor 594-labeled goat anti-mouse IgG and anti-rabbit IgG (all from Abcam; 1:1000) and Alexa Fluor 647-labeled donkey anti-rabbit IgG (Life Technology; 1:1200). Nuclei were counterstained with Hoechst 33342 (Sigma), and coverslips were mounted in mounting medium (made in house).

### Fluorescence *in situ* hybridization

Cells were grown on coverslips and fixed for 20 min in 4% PFA and overnight with 70% ethanol at 4°C. The next day, cells were washed with PBS and treated for 2.5 min with 0.5% Triton X-100. Cells were washed with PBS and incubated for 10 min in 15% formamide (in 4% SSC; Bio-Lab Ltd, Jerusalem, Israel). Cells were hybridized overnight at 37°C in 15% formamide with a specific Cy3-labeled oligo(dT) DNA probe (∼10 ng probe, 50mer). The next day, cells were washed twice with 15% formamide for 15 min and then immunofluorescence was performed after the RNA FISH using the standard protocol described above. Nuclei were counterstained with Hoechst 33342, and coverslips were mounted in mounting medium.

smFISH experiments with Stellaris probes (Biosearch Technologies) were performed according to the manufacturer's adherent cell protocol for MKI67 and IPO7 mRNA, as previously described (Sheinberger et al., 2017). NORAD probes were from Igor Ulitsky (Weizmann Institute of Science, Rehovot, Israel; Zuckerman et al., 2020). To reduce photobleaching, cells were submerged in GLOX buffer (pH 8, 10 mM Tris-HCl, 2× SSC, 0.4% glucose) supplemented with 3.7 ng of glucose oxidase (Sigma, G2133-10KU) and 1 µl catalase (Sigma, 3515) prior to imaging (Raj et al., 2008; Yildiz et al., 2003).

### Fluorescence microscopy, live-cell imaging and data analysis

Wide-field fluorescence images were obtained using either the CellSens system based on an Olympus IX83 fully motorized inverted microscope (60× UPlanXApo objective, 1.42 NA) fitted with a Prime BSI sCMOS camera (Teledyne) driven by the CellSens software; or with the Cell^R/Scan^R system based on an Olympus IX81 fully motorized inverted microscope (60× PlanApo objective, 1.42 NA) fitted with an Orca-AG CCD camera (Hamamatsu) driven by the Cell^R software. Live-cell imaging was carried out using the Cell^R system with rapid wavelength switching. For time-lapse imaging, cells were plated on glass-bottomed tissue culture plates (MatTek, Ashland, MA) in medium containing 10% FBS at 37°C. The microscopes were equipped with an incubator that includes temperature and CO_2_ control (Life Imaging Services, Reinach, Switzerland).

Confocal imaging was performed on a Leica SP8 inverted microscope equipped with a pulsed white-light laser and gating. The objective used was a 100×1.4 NA, with Leica immersion oil, at room temperature. Organoids were imaged on a confocal LSM700 ZEISS microscope, using a 40× oil lens, NA 1.518. Movies were deconvolved using Huygens Essential II with a time-series option (Scientific Volume Imaging, Hilversum).

For counting the number of SG-positive cells in a cell population, SGs were counted manually, with at least 100 cells per treatment, using ImageJ software. For counting of cells positive for mitotic catastrophe in U2OS wild-type and ΔΔG3BP1/2 cells, more than 14 fields of cells were imaged using an Olympus IX83 fully motorized inverted microscope, and stitching of the fields was performed using the CellSens software. Over 80 cells were counted per stitched field (and counting was performed with *n*=3). Mitotic catastrophe-positive cells were identified by the appearance of multi-nucleated cells.

### Fluorescence recovery after photobleaching

U2OS cells were maintained in low DMEM medium (Biological Industries). Images were acquired using a DMi8 Leica wide-field inverted microscope (Leica Microsystems, Mannheim, Germany) equipped with an Infinity scanner with standard laser lines, an sCMOS DFC9000GT Leica camera and a 63×1.4 NA objective. The microscope was driven using LasX software (Leica). Three cell types were analyzed: (1) U2OS cells stably expressing β-actin mRNA with MS2 stem-loops (Ben-Ari et al., 2010) and the YFP–MS2-A1 coat protein (Mor et al., 2010), which were activated with doxycycline for 24 h before treatment; (2) U2OS cells stably expressing GFP–IGF2BP3; and (3) U2OS cells that were transiently transfected with G3BP1–GFP (Marmor-Kollet et al., 2020) 24 h before activation using the PolyJet reagent, then activated for 24 h before induction using doxycycline. Cells were treated with 75 µM VRB, 75 µM VRB with 300 µM cortisone, or 0.5 mM sodium arsenite (Sigma). For rinses, cells were treated with either 75 µM VRB or 75 µM VRB with 300 µM cortisone, then were rinsed once with fresh medium before being left in fresh medium.

After image acquisition parameter setup, regions of interest (ROIs) were chosen, and the 515 nm laser for mRNA (YFP–MS2) or the 488 nm laser for GFP-tagged proteins were set to maximum power for ROI bleaching. Five images were acquired pre-bleach, and then 70 images were acquired post-bleach at 3 s intervals. Images were then processed within the LasX program as part of the FRAP workflow. In some cells, multiple SGs were bleached per cell to accommodate the naturally wide variance in SG fluorescence. The data were analyzed as previously described (Shav-Tal et al., 2005). Briefly, background was subtracted from each time point, followed by normalization for natural photobleaching during acquisition using measurements taken from an ROI in an unbleached region of the cytoplasm. Values were then normalized based on the initial image's pre-bleach intensity and the natural photobleaching to provide the relative intensity of the recovery. Curves represent the average of replicates, shown alongside the line of best fit. For *n* of bleached SGs in VRB treatment (*n*_VRB_), VRB and cortisone treatment (*n*_VRB+Cor_), arsenite treatment (*n*_Arsenite_), VRB washout (*n*_VRB wash_), and VRB and cortisone washout (*n*_VRB+Cor wash_): β-actin MS2 curves, *n*_VRB+Cor_=123, *n*_VRB_=118; G3BP1 curves, *n*_VRB+Cor_=124, *n*_VRB_=109, *n*_Arsenite_=38; IGF2BP3 curves, *n*_VRB+Cor_=216, *n*_VRB_=159, *n*_VRB wash_=71, *n*_VRB+Cor wash_=68.

Three-parameter asymptotic regression was used to analyze FRAP experiments. Each replicate was fitted to a curve defined by the equation *Y*=*a*−(*a*−*b*) e^(−c*X*)^ where *X* is time and *Y* is the relative intensity, *a* (plateau) is the maximum attainable intensity, *b* (init) is the initial *Y* value (at time=0) and *c* (m) is proportional to the relative rate of increase for intensity when time increases. The regression fitting was performed using the *drm* function from the *drc* R package (Ritz et al., 2015) and *DRC.asymReg* self-starting function from the *aomisc* R package (Onofri et al., 2021; https://www.statforbiology.com).

Each parameter (init, m and plateau) was then compared between all treatments with a one-way nested ANOVA, followed by pairwise comparisons of mean values between treatments. Finally, *P*-values were adjusted for multiple comparisons with the Benjamini–Hochberg (false discovery rate, FDR) procedure. The function *lmer* from *lmerTest* R package (Kuznetsova et al., 2017) and *emmeans* from *emmeans* R package (emmeans: Estimated Marginal Means, aka Least-Squares Means, R package version 1.7.1-1; https://CRAN.R-project.org/package=emmeans) were used.

### Cell viability

Cells were treated with VRB (10 µM), cortisone (300 µM) or methanol (3%) for 24, 48 and 72 h. Dead cells were collected as well as live cells after trypsinization, and samples were centrifuged at 1000 rpm (200 ***g***) for 5 min. Cells were suspended in binding buffer and stained with annexin V–FITC and PI using the MEBCYTO Apoptosis kit (MBL). Flow cytometry was performed on a BD LSR Fortessa Cell Analyzer. Analysis was performed using FlowJo Ver 10.8 software. Cell viability was determined by the population negative for annexin V and PI.

### Statistical analysis

Experiments presented were repeated at least three times. All statistical analyses were performed using R statistical software (https://www.R-project.org/).

For quantification of SG-positive cells in various cell types, data were analyzed with two-tailed independent sample *t*-tests (in the case of two groups) or with a one-way ANOVA followed by Tukey's post hoc analysis. Treatment groups for which all values were constant (0% cells) were analyzed separately from other treatments using two-tailed one-sample *t*-tests against a constant mean value of 0. Finally, an FDR correction (Benjamini–Hochberg method) was applied to adjust for multiple testing.

For the organoids, a Fisher's exact test was used to test for presence of SGs under different conditions. Specifically, experiments with and without SGs were counted, and proportions were compared between conditions. Post hoc analysis was performed as pairwise testing between conditions, and *P*-values were adjusted for multiple comparisons with the Benjamini–Hochberg (FDR) procedure.

For the live-cell assays: in the VRB 50 µM replicates, a two-tailed one-sample *t*-test against a constant mean value of 0 was used to compare the fraction of SG-positive cells of two treatments at the last time point; and in the VRB 75 µM rinse replicates, exponential decay regression was used to analyze experiments. Each experiment was fit to a curve defined by the equation *Y*=*ae^−kX^* where *X* is time, *Y* is relative intensity, *a* (init) is the initial *Y* value (at time=0) and *k* (m) is the exponential decay rate representing the relative decrease of *Y* for a unit increase of *X*. The regression fitting was performed using the *drm* function from the *drc* R package and the *DRC.expoDecay* self-starting function from the *aomisc* R package. The rate parameter was then compared between two treatments using a two-tailed independent sample *t*-test.

For the mitotic catastrophe and annexin assays, data were analyzed with a two-way ANOVA. Post hoc analysis was performed as pairwise comparisons defined by linear contrasts, Finally, *P*-values were adjusted for multiple comparisons with the Benjamini–Hochberg (FDR) procedure.

## Supplementary Material

Supplementary information

Reviewer comments
