# Supplementary material for: Glucocorticoids enhance chemotherapy-driven stress granule assembly and impair granule dynamics, leading to cell death
Source: J Cell Sci. 2022 Jul 26;135(14):jcs259629. doi: 10.1242/jcs.259629 (PMC9450892; doi:10.1242/jcs.259629)
Supplement: Supplementary information [file joces-135-259629-s1.pdf]

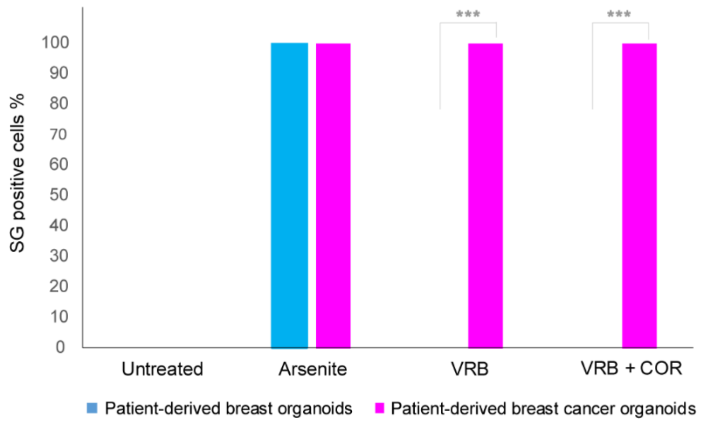

Fig. S1. VRB and cortisone induce the formation of SGs in patient-derived breast cancer organoids. Quantification of the population of SG-positive cells when cortisone was added to VRB or Arsenite treatments, in patient-derived breast organoids originating from either healthy tissue or from cancerous tissue. Data were analyzed using Fisher's Exact Test (***p<0.001), n=30 organoids.

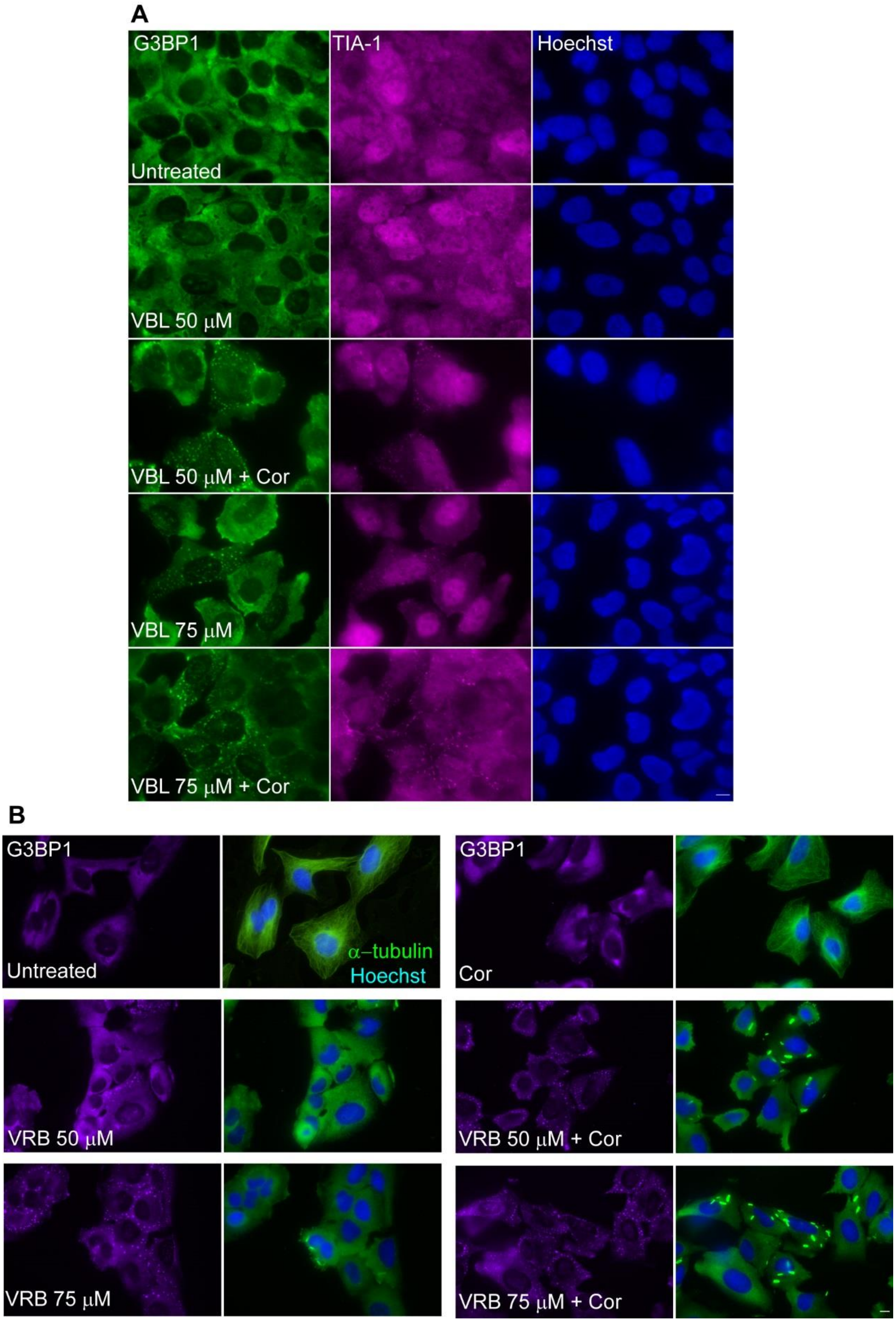

Fig. S2. Cortisone enhances SG formation by the related microtubule inhibitor vinblastine. (A) The formation of SGs in U2OS cells under vinblastine (VBL; 50-75 µM) and Cor (300 µM) for 1 hr was detected using anti-G3BP1 (green) and anti-TIA1

(magenta) SG markers. Hoechst DNA stain is in blue. Bar = 10 µm. (**B**) The formation of SGs in U2OS cells under VRB (50-75 µM) and Cor (300 µM) for 1 hr coincides with the breakdown of the microtubule network (α-tubulin; green) and the formation of tubulin para-crystals (green rods). G3BP1 (magenta) was used as the SG marker. Hoechst DNA stain is in blue. Bar = 10 µm.

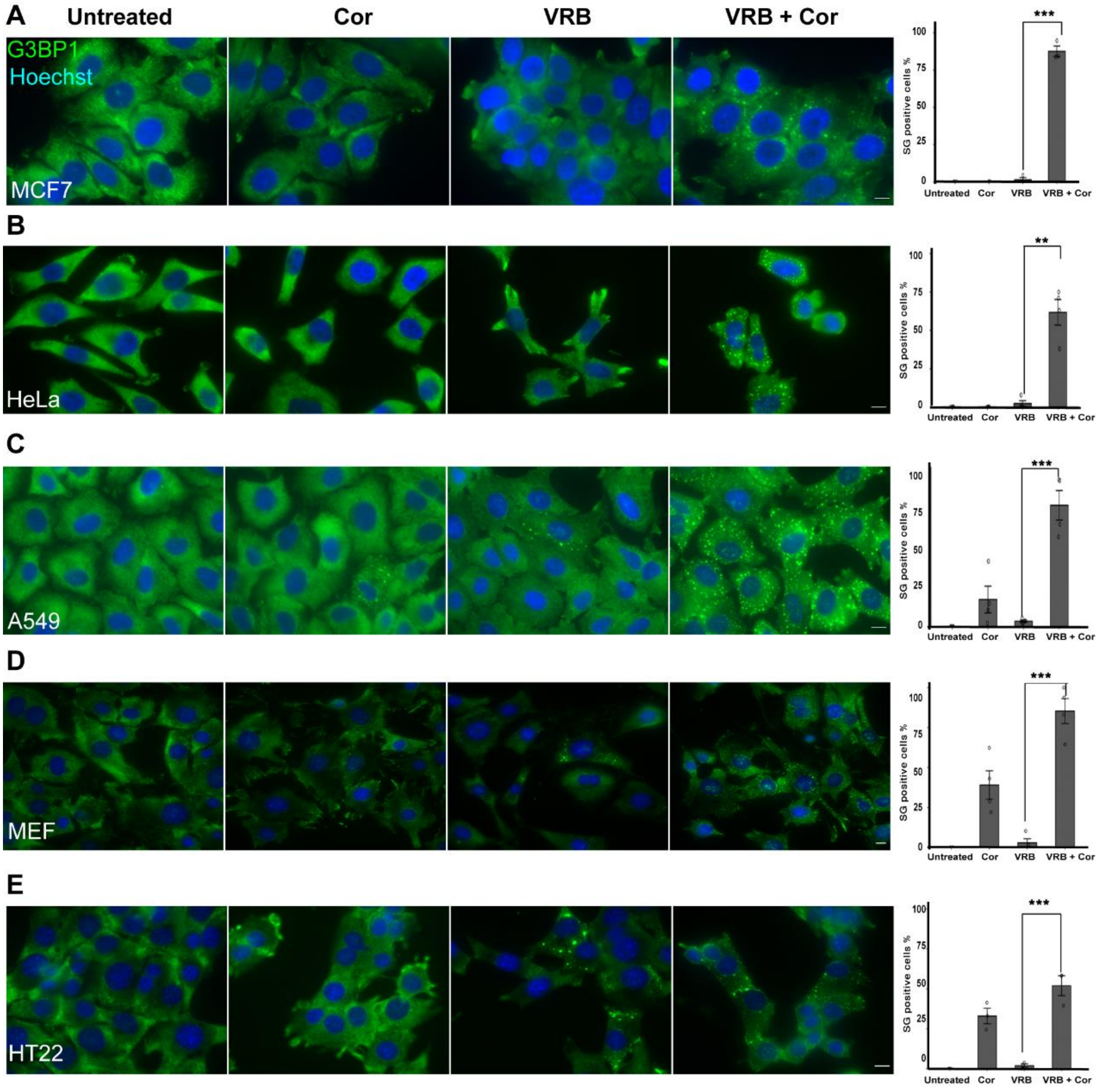

Fig. S3. The formation of SGs in different cell lines. Quantification of the population of SG-positive cells under the different treatment conditions. **(A)** SG formation under VRB (75 µM) + Cor (300 µM) for 1 hr in MCF7 cells (n=90 cells per treatment). **(B)** SG formation under VRB (30 µM) + Cor (300 µM) for 1 hr in HeLa cells (n=160 cells per treatment). **(C)** SG formation under VRB (75 µM) + Cor (300 µM) for 1 hr detected in A549 cells (n=149 cells per treatment). **(D)** SG formation under VRB (15 µM) and Cor (300 µM) for 1 hr in a MEF cell line (Sullivan et al., 1999) (n=123 cells per treatment). **(E)** SG formation under VRB (15 µM) and Cor (300 µM) for 1 hr in HT22 cells (n=139 cells per treatment). SGs were detected using anti-G3BP1 (green). Hoechst DNA stain is in blue. Bar = 10 µm. Data were analyzed using the 1-way ANOVA + Tukey's post hoc and 1-sample t-tests against m=0 and m=100 (**p<0.01, ***p<0.001), n=3. Bar graph illustrates the mean and standard deviation (STDEV, error bars). Each circle on the bar graph indicates a biological replicate.

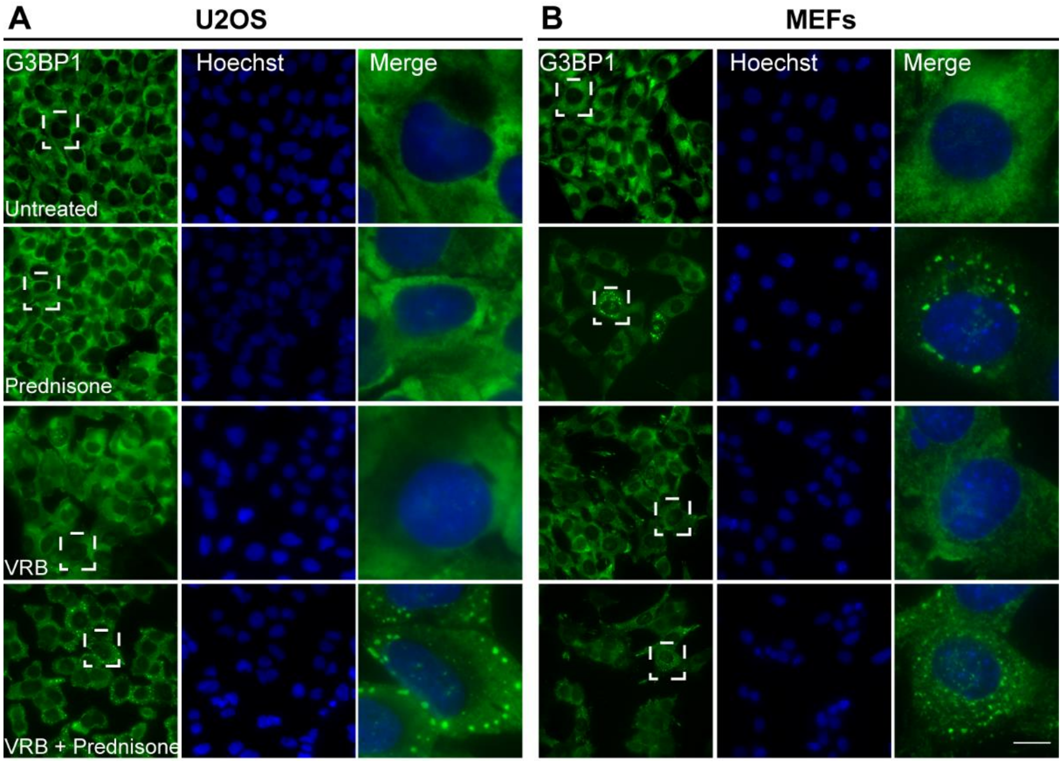

Fig. S4. Prednisone enhances the formation of VRB-induced SGs. (A) The formation of SGs under VRB (50 µM) and Prednisone (Pre; 300 µM) for 1 hr in U2OS cells was detected using the anti-G3BP1 (green) SG marker. Hoechst DNA stain is in blue. **(B)** The formation of SGs under VRB (15 µM) and Prednisone (Pre; 300 µM) for 1 hr in MEF cells was detected using anti-G3BP1 (green). Hoechst DNA stain is in blue. Enlargements of the boxed areas appear on the right. Bars = 10 µm.

Journal of Cell Science • Supplementary information

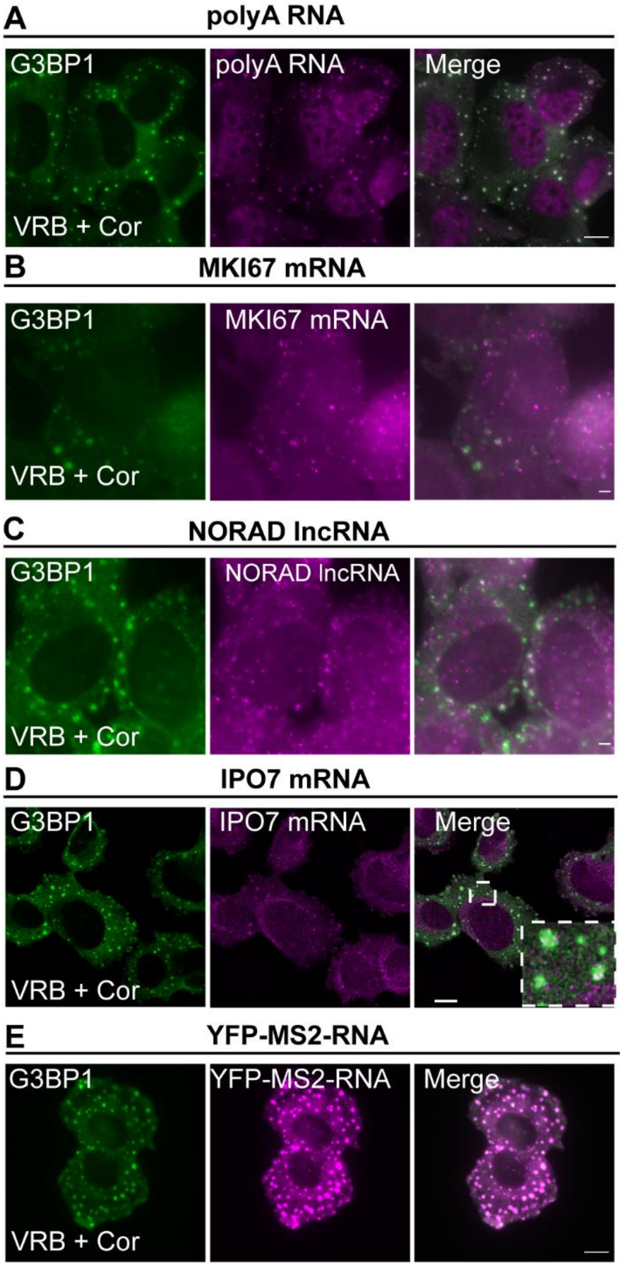

Fig. S5. VRB and cortisone induce the accumulation of RNA in SGs. (A) VRB + Cortisone treatment affects the sub-cellular distribution of poly(A)+ RNA in U2OS cells. U2OS cells treated with VRB (75 µM) +Cor (300 µM) for 1 hr show the localization of poly(A)+ RNAs, detected by RNA FISH with an oligo-dT fluorescent probe (magenta), into SGs labeled with anti-G3BP1 (green). **(B-E)** The localization of endogenous transcripts (magenta) to SGs (anti-G3BP1, green) was examined by RNA FISH for (B) MKI67 mRNA; (C) NORAD lncRNA; (D) IPO7 mRNA; (E) β-actin mRNAs (YFP-MS2-CP, magenta; Dox-induced overnight); after VRB (75 µM) +Cor (300 µM) treatments for 1 hr. Images were acquired on a confocal microscope. Boxed region is shown in the enlarged image.

Journal of Cell Science • Supplementary information

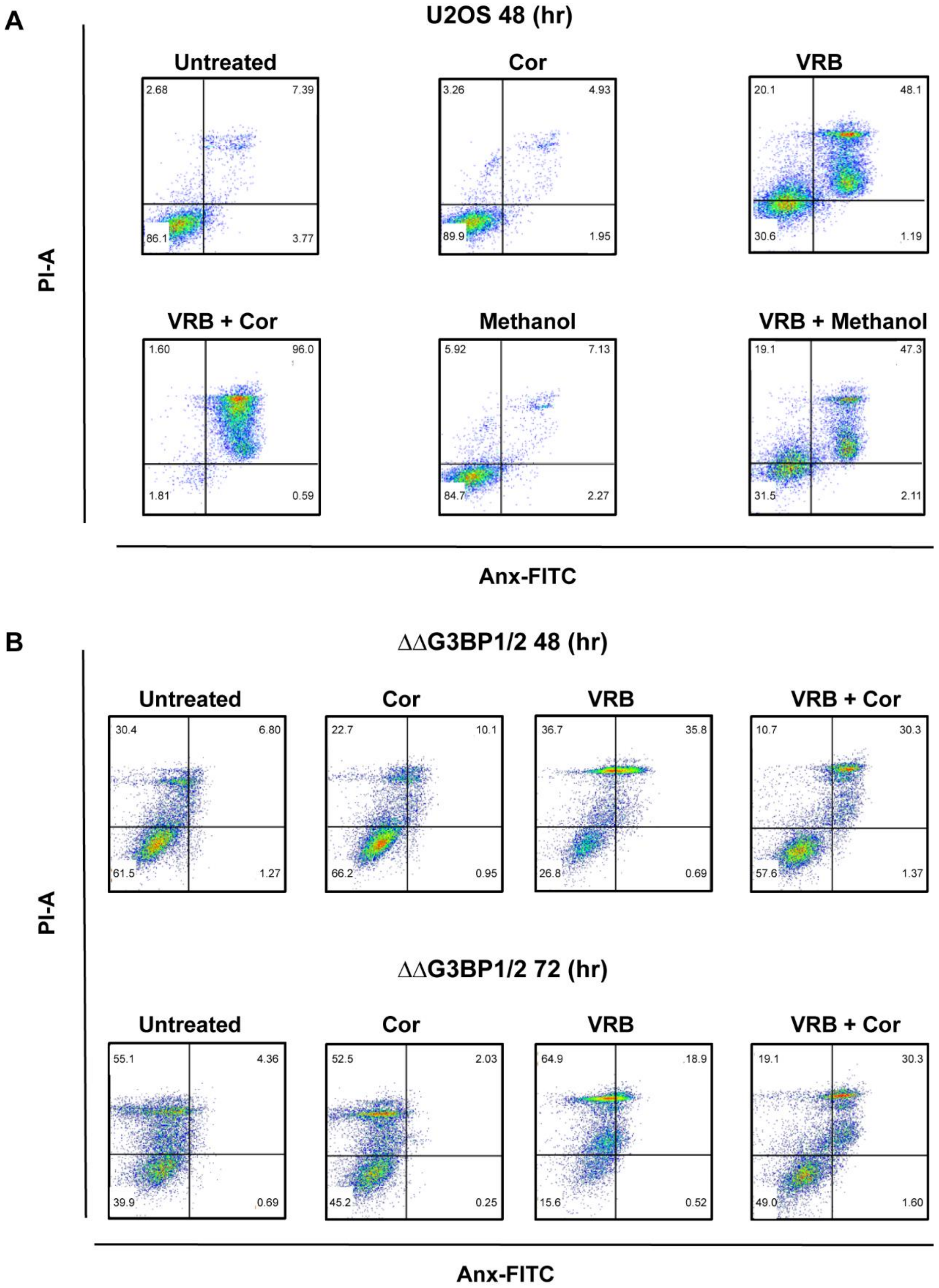

Fig. S6. Cortisone affects the viability of cells treated with VRB. Annexin V and propidium (PI) analysis was performed on U2OS and ΔΔG3BP1/2 cells treated with VRB (10 µM), cortisone (300 µM) and methanol (3%) for 48 and 72 hrs, to monitor cell

Journal of Cell Science • Supplementary information

death under these conditions. **(A)** Two-dimensional dot plots of the different subpopulations including methanol controls as detected by flow cytometry data at 48 hrs are presented. Numbers in each quadrant represent the percentage of cells from the population detected. **(B)** Two-dimensional dot plots for the ΔΔG3BP1/2 cells treated with VRB (10 µM) and cortisone (300 µM) at the 48 and 72 hrs time points.

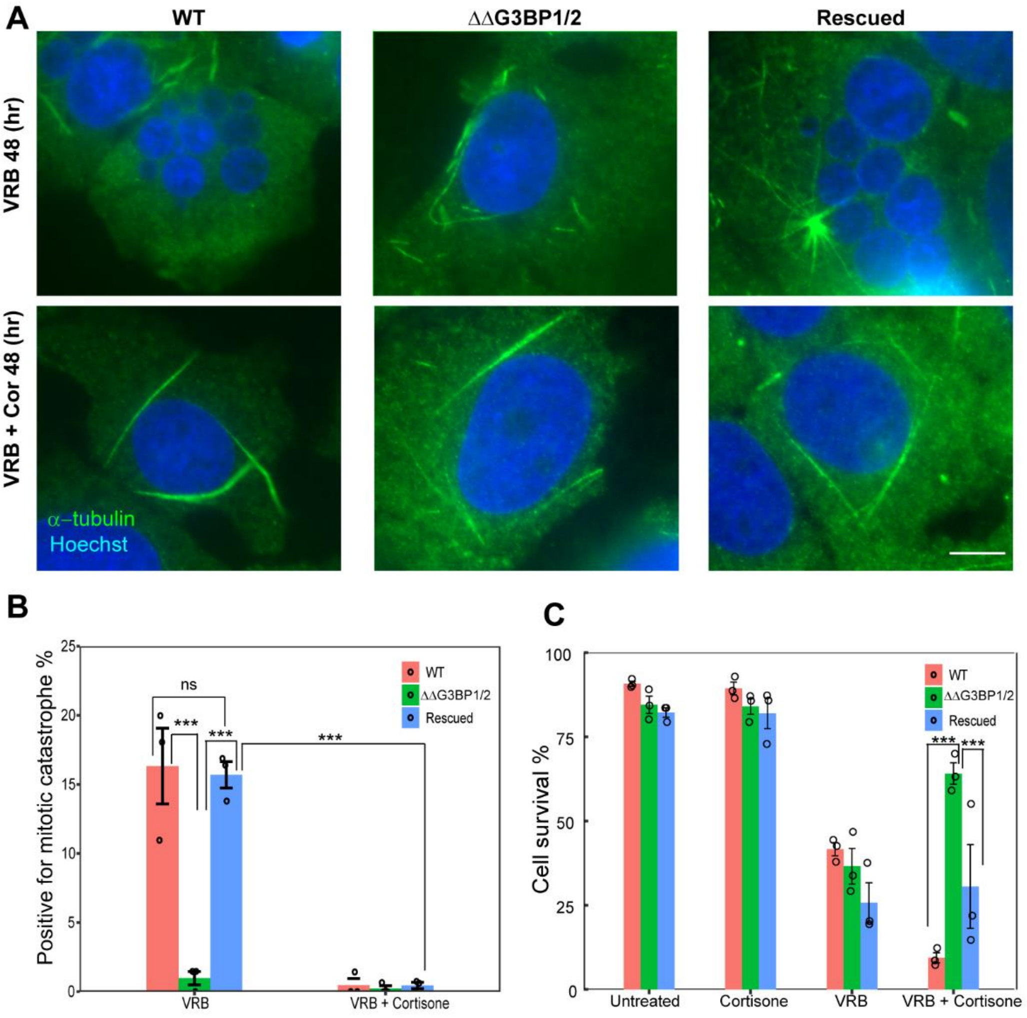

Fig. S7. G3BP1 rescues the effects seen in the ΔΔG3BP1/2 cells. (A) U2OS wildtype, ΔΔG3BP1/2 and G3BP1-rescued-ΔΔG3BP1/2 cells were treated with VRB (10 μM) and cortisone (300 μM) for 48 hrs and stained with α-tubulin (green). Mitotic catastrophe was observed by Hoechst DNA staining (blue). Bar=10 μm. **(B)** Quantification of positive U2OS, ΔΔG3BP1/2 and G3BP1-rescued-ΔΔG3BP1/2 cells for mitotic catastrophe treated with VRB (10 μM) and VRB+cortisone (300 μM) for 48 hrs (n=3) (ns- non significant, ***p<0.001). **(C)** Graphical representation of Annexin V PI results in U2OS, ΔΔG3BP1/2 and G3BP1-rescued-ΔΔG3BP1/2 cells, showing the percentage of live cells (Annexin V and PI negative). U2OS, ΔΔG3BP1/2 and G3BP1-rescued-ΔΔG3BP1/2 cells were treated simultaneously with VRB (10 μM) and cortisone (300 μM) for 48 hrs. Data were analyzed by 2-way ANOVA following Tukey's post hoc (***p<0.001).

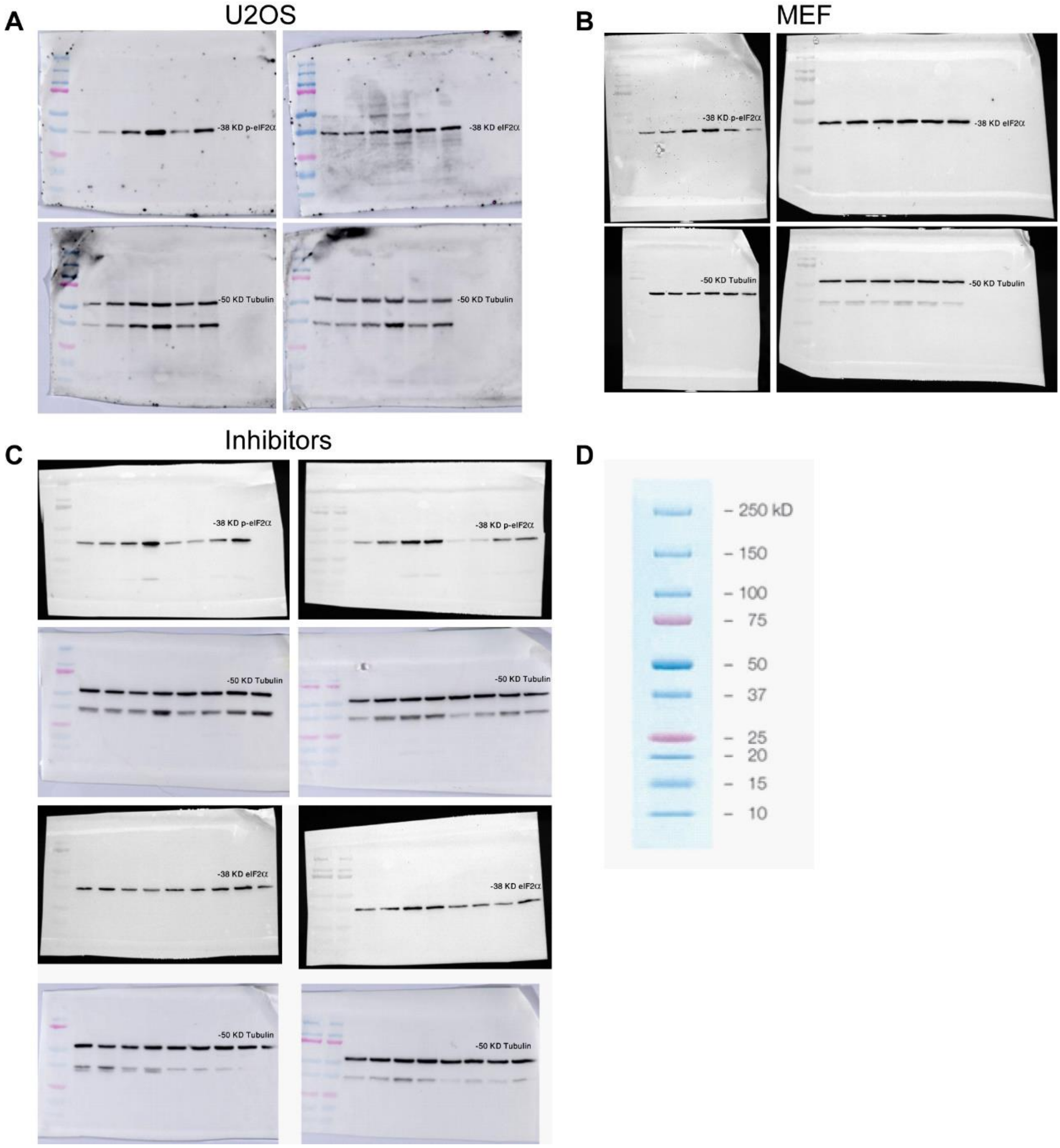

Fig. S8. Blot Transparency. Blots from Western blot analysis corresponding to main figures: **(A)** Fig. 2C; **(B)** Fig. 6C; **(C)** Fig. 8D. **(E)** Protein ladders (Bio-Rad).

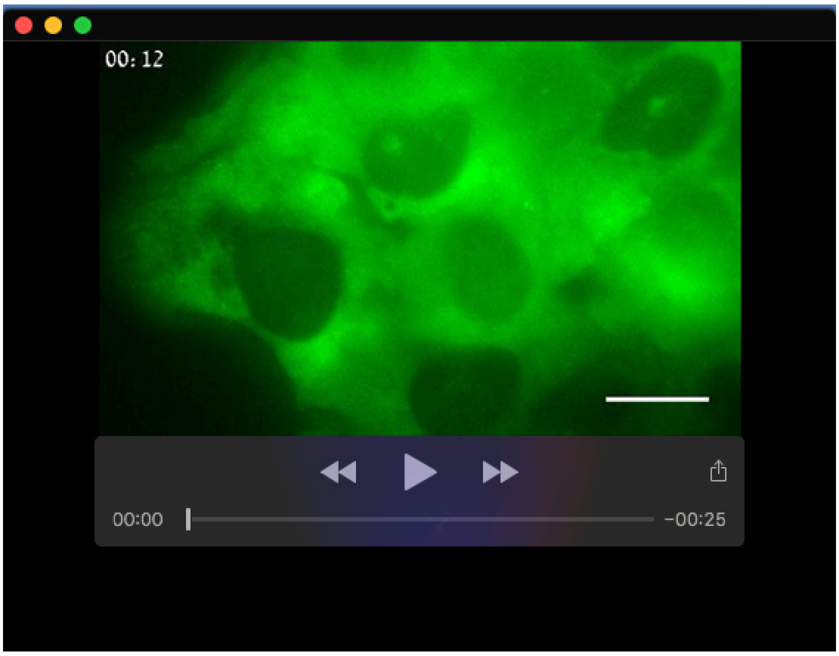

Movie 1. Live cell imaging of SGs in U2OS cells stably expressing GFP-IGF2BP3 under treatment with VRB (50 µM). Images were acquired every 3.5 minutes for 68.5 min. Cells treated with VRB showed no SGs.

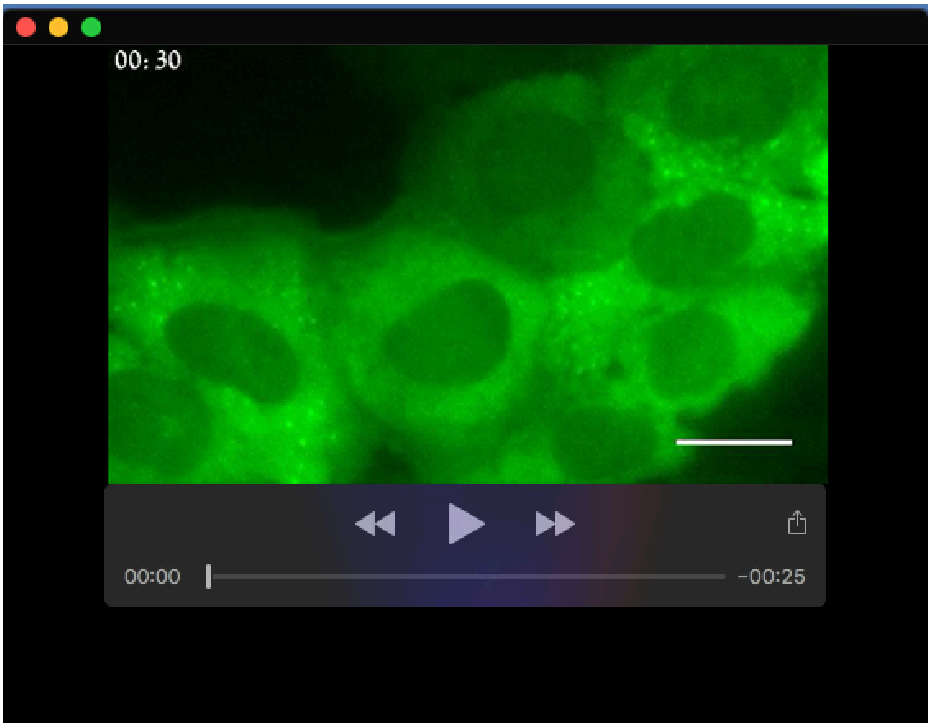

Movie 2. Live cell imaging of SGs in U2OS cells stably expressing GFP-IGF2BP3 under treatment with VRB (50 µM) and cortisone (300 µM). Images were acquired every 3.5 minutes for 68 min. Cells treated with VRB and cortisone formed SGs.

Journal of Cell Science • Supplementary information

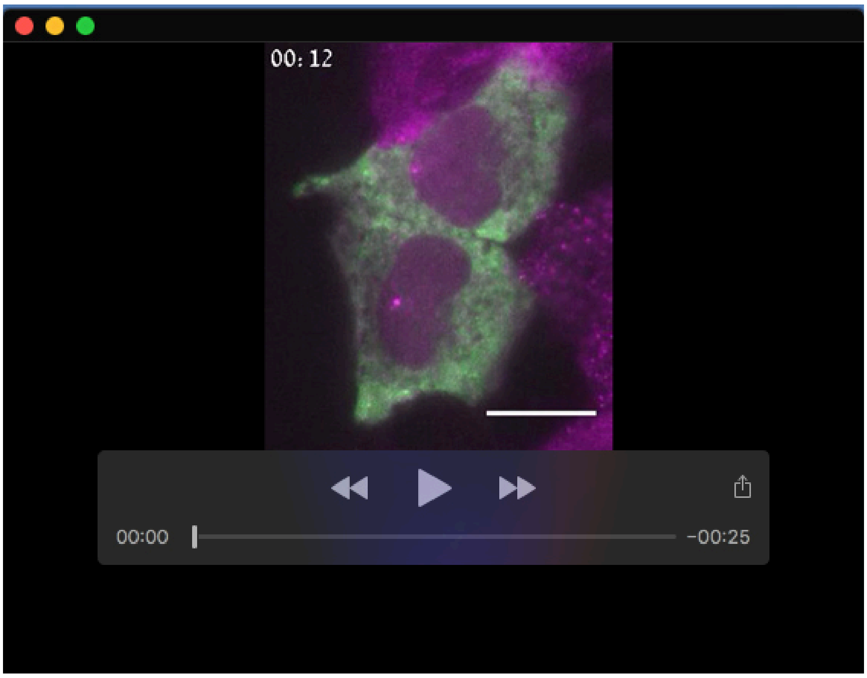

Movie 3. Live-cell imaging of SGs in U2OS cells stably expressing β-actin mRNA and YFP-MS2-CP, along with transient transfection of mCherry-IGF2BP3, under treatment with VRB (75 µM). Images were acquired every 5 minutes for 72 min.

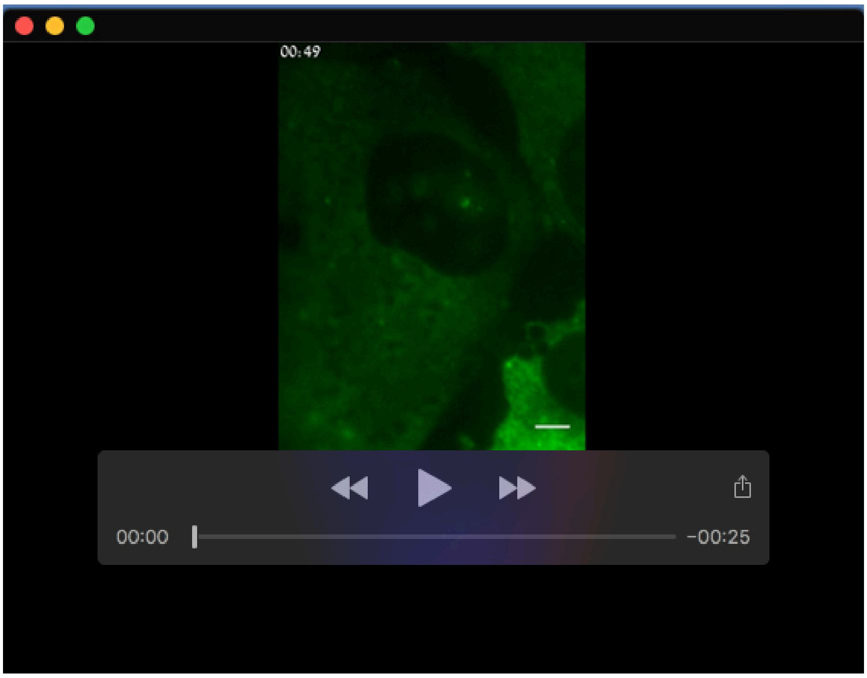

Movie 4. Live-cell imaging of SGs in U2OS cells stably expressing GFP-IGF2BP3 treated with VRB 75 µM for 1 hr, and then washed with fresh medium. Images were acquired every 3.5 min for 68.5 min. SGs dissipated after ~30 min under VRB treatment.

Journal of Cell Science • Supplementary information

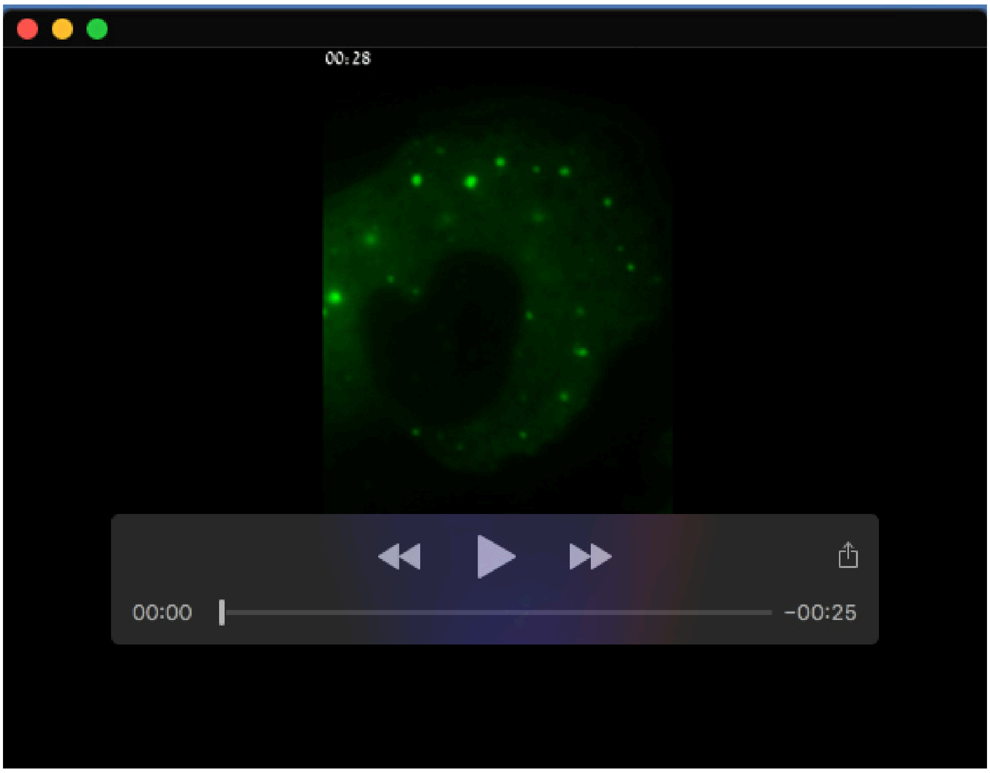

Movie 5. Live-cell imaging of SGs in U2OS cells stably expressing GFP-IGF2BP3 treated with VRB 75 µM and cortisone 300 µM for 1 hr, and then washed with fresh medium. Images were acquired every 3.5 min for 68.5 min. SGs were still present after 1 hr after rinse when the treatment included cortisone.
