# Supplementary material for: Glucocorticoids enhance chemotherapy-driven stress granule assembly and impair granule dynamics, leading to cell death
Source: J Cell Sci. 2022 Jul 26;135(14):jcs259629. doi: 10.1242/jcs.259629 (PMC9450892; doi:10.1242/jcs.259629)
Supplement: Reviewer comments [file joces-135-259629_review_history.pdf]

Glucocorticoids enhance chemotherapy-driven stress granule assembly and impair granule dynamics, leading to cell death

Avital Schwed-Gross, Hila Hamiel, Gabriel P. Faber, Mor Angel, Rakefet Ben-Yishay, Jennifer I. C. Benichou, Dana Ishay-Ronen and Yaron Shav-Tal
DOI: 10.1242/jcs.259629

Editor: Maria Carmo-Fonseca

Review timeline

Original submission:	24 November 2021
Editorial decision:	10 January 2022
First revision received:	26 May 2022
Accepted:	13 June 2022

Original submission

<u>First decision letter</u>

MS ID#: JOCES/2021/259629

MS TITLE: Glucocorticoids enhance chemotherapy-driven stress granule assembly and promote granule rigidity leading to cell death

AUTHORS: Avital Schwed-Gross, Hila Hamiel, Gabriel P Faber, Mor Angel, Rakefet Ben-Yishay, Dana Ishay-Ronen, and Yaron Shav-Tal
ARTICLE TYPE: Research Article

Dear Yaron,

We have now reached a decision on the above manuscript.

To see the reviewers' reports and a copy of this decision letter, please go to: https://submit-jcs.biologists.org and click on the 'Manuscripts with Decisions' queue in the Author Area. (Corresponding author only has access to reviews.)

As you will see from their reports, the reviewers' recommendations regarding publication are somewhat mixed. While referee #2 considers that your study represents too little progress for warranting publication, the other two thought that the work was potentially quite interesting and significant but all also raised a number of concerns that must be dealt with. Please address the issues raised by the three reviewers as thoroughly as possible. If you think that you can deal satisfactorily with the criticisms on revision, I would be pleased to see a revised manuscript. I would then return it to the reviewers.

We are aware that you may be experiencing disruption to the normal running of your lab that makes experimental revisions challenging. If it would be helpful, we encourage you to contact us to discuss your revision in greater detail. Please send us a point-by-point response indicating where you are able to address concerns raised (either experimentally or by changes to the text) and where you will not be able to do so within the normal timeframe of a revision. We will then provide further guidance. Please also note that we are happy to extend revision timeframes as necessary.

© 2022. Published by The Company of Biologists under the terms of the Creative Commons Attribution License (https://creativecommons.org/licenses/by/4.0/).

Please ensure that you clearly highlight all changes made in the revised manuscript. Please avoid using 'Tracked changes' in Word files as these are lost in PDF conversion.

I should be grateful if you would also provide a point-by-point response detailing how you have dealt with the points raised by the reviewers in the 'Response to Reviewers' box. Please attend to all of the reviewers' comments. If you do not agree with any of their criticisms or suggestions please explain clearly why this is so.

Reviewer 1

Advance summary and potential significance to field

In their study Schwed-Gross et al. investigate the dynamic formation of stress granules (SGs) and SG-association with stress signaling pathways as well as cell viability upon treatment with cortisol and/or chemotherapeutics of the vinca-alkaloid family, specifically vinorelbine (VRB). The authors present very interesting findings, including one of the first investigations of these compounds in cancer organoids.
Findings by Schwed-Gross et al., reveal that VRB- and cortisol-dependent induction of SGs is eIF2a-dependent, but relies on partially distinct upstream signaling, PERK- vs. GCN2-modulated pathways. Most interestingly, the authors show a strong cell type-dependence of their findings and distinct protein/RNA-dynamics in/at SGs upon VRB/cortisol treatment. The study is of particular interest for the cancer field, since SG formation was observed exclusively in patient-derived breast cancer organoids, but not non-malignant breast organoids. Although the study remains largely descriptive it provides highly valuable information for further exploration of the role(s) SGs may serve in the clinical management of cancer by chemotherapy. The presented manuscript is well-written, although the authors may want to consider shortening the introduction and discussion where appropriate. In sum, the presented study provides valuable, high-quality insights which set the stage to further explore the highly complex role of SGs and G3BPs in diseases, in particular cancer.

Comments for the author

Minor aspects to address:
1. If feasible, it would be interesting to investigate how eIF2a abundance and phosphorylation is affected in non-malignant vs. patient-derived breast cancer organoids. This may provide insights on the discussed influence of eIF2a abundance on malignant transformation or therapy resistance, respectively. Moreover, can the authors provide statistics on SG formation in organoids (Fig.3)?
2. Can the authors address or speculate why cortisol shows potentially distinct effects on SG dynamics in distinct cells, in particular in primary cancer organoids. May this be associated with eIF2a abundance or upstream signaling components?
3. Can the authors address or speculate (beyond rigidity) on the mechanisms behind the distinct protein dynamics observed in cortisol-induced SGs and why more rigid SGs may promote cell death?
4. Most studies were performed at VRB concentrations ~100-fold higher than expected/reported (https://www.cancerrxgene.org/) IC50 values for the respective cell lines. Do the cells form SGs when treated for longer time (72h) at low doses of VRB +/- cortisol?
5. Minor typo: L65, instead of IGFBPs this should read IGF2BPs
6. The authors should check if the used cell lines are indicated in each figure legend to support data evaluation.

Reviewer 2

Advance summary and potential significance to field

This MS shows that cortisone enhances the formation of SG triggered by exposure to Vinorelbine (VRB), an anti-cancer drug that acts on microtubule polymerization. Enhanced SG induction correlated with enhanced P-2alpha (as it could be expected, as the integrated stress response is typically accompanied by SG formation). In addition, authors propose that cortisone modulates the

© 2022. Published by The Company of Biologists under the terms of the Creative Commons Attribution License (https://creativecommons.org/licenses/by/4.0/).

biophysical properties of VRB-induced SGs driving a transition towards pro-death SGs. This is an overinterpretation. The combination of cortisone plus VRB induced serious cell damage, and as a consequence all stress markers resulted enhanced, including eIF2alfa phosphorylation (which is known can induce apoptosis) as well as SG formation and integrity. The advance is rather scarce.

Comments for the author

The work is mainly descriptive and lacks mechanistic insights. A number of quantifications and important controls are missing. In addition, the main conclusion is not supported by the data, as follows.
1. QUANTIFICATIONS IN Figure 1A, 1B, S4, S5, S6, S7 and S8 Showing a single image for each condition/staining is not acceptable. SG formation should be analyzed in a significant number of cells in several replicate. (Data in panel 1A can be omitted without substantial loss as this is not a novel observation and is repeated in other figures)
2. FIGURE 1A:
a) eIF3B is known to be mostly cytoplasmic in U2OS cells. Is there a reason for the strong nuclear signal shown here?
b) P-2alpha: its presence in puncta is not clear. Is this a bonafide SG marker?
3. Figure 1B a) As above, quantifications are strictly required. Showing a single example for each condition is not acceptable. How many healthy and tumor-derived organoids were analyzed, and how many cells in each sample? Were patients under any kind of treatment? Healthy and tumoral tissue from the same patient will work better.
b) Line 178 and 222: data certainly do not show “the potency of VRB action on cancer cells”
4. FIGURE 2
a) FIGURE 2: In the “100 uM VRB plus Cor” panel, SGs are not apparent in the enlarged cell.
b) In addition to the number of cells with SGs, quantification of the number of SG per cell and their size may help to describe the response. For example, SG seems larger upon 75uM VRB plus Cor than upon 75uM VRB alone. This may be in connection with a stronger ISR and stronger eIF2alpha phosphporylation.
c) line 187: I agree that cell detachment is a sign of cell damage, suggesting that the insult is not a sublethal stress (see point 7 below).
d) Figure 2D and E, and Figure S9 (videos). SG formation and dissolution should be quantitated at several time points and independent replicates should be analyzed. Again, the number of cells with SGs is a binary readout that limits the analysis.
e) The initial distribution of IGF2BP3 in the cells shown in S9 is not comparable. The lower cell shows a greater intensity ratio SGs/cytosol, and it is expected that it will take longer for this signal to dissipate. See point d above.
f) Finally and very important, observations in 2D and Eand S9 are redundant with the above results: if more cells show SGs at 60 min (panel 2B) is expected that more cells will show SGs at earlier time points (30 min) and that more cells will show SGs at later times, during recovery. And all this correlates with enhanced 2alphaP, as expected. It would be more clear if all these observations are presented as readouts of the same effect, rather than as independent effects of the treatment.
5. FIGURE 4: what do we learn from this? What is the relevance of describing the effect in mouse cells? Another example of descriptive work that lack mechanistic insights.
6. FIGURE 5F: G3BP1 FRAP. G3BP1 is a key SG assembly factor and condense in the so-called SG cores. Thus, it is expected that G3BP1 exchange with the cytosol will be affected by the strength of the stress and the concomitant increase in SG formation (see point 3.e above). In other words, how specific is the observed effect for the VRB plus COR treatment? Is the nature of the stressor or the strength of the ISR the factor that affect SG dynamics? Does it change along the time-course of SG formation? Does it depend on the SG size? Is this change linked to the 2alpha kinase that is being activated (cortisone induces GCN2 and VRB induces PERK)? Investigating some of these questions will help understanding the reason of the observed change.
Finally, does it make sense that mRNA exchange is not altered and at the same time protein exchange is affected? It will be nice to elaborate on this.
7. The use of G3BP1/2 KO cells: The effect of the recovery G3BP1/2 levels should be analyzed. KO cells should be transfected with G3BP 1/2 and all measurements repeated. Phenotype rescue is an important control strictly required to conclude that the effects reported are specifically due to lack of G3BPs.

© 2022. Published by The Company of Biologists under the terms of the Creative Commons Attribution License (https://creativecommons.org/licenses/by/4.0/).

8. The main conclusion: Authors propose that cortisone changes the biophysical properties of VRB-induced SGs and that this change the function of SGs from a protective role to a cell-death inducing role. This is not an immediate conclusion. Increased cell death may be linked to the stronger insult that results from the combined treatment. VRB activates the PERK pathway (how?) and cortisone activates GCN2 (how?). When both 2alpha kinases are activated the response is stronger, shifting from a sub-lethal to a more lethal stress. P-2alpha levels are higher (which is known to induce pro-apoptotic factors, CHOP among others) and SG formation is enhanced (with several parameters being affected, including reduced dynamics of SG core proteins). The data are insufficient to conclude that VRB-induced SGs are protective whereas SGs induced by VRB + cortisone are pro-death. Rather, SGs are a consequence and a marker of the cell reaction and the survival/death balance is not directly influenced by them. The effect of ISRIB shown here, which inhibits the translational silencing downstream of P-2alpha and concomitant SG formation, as clearly summarized in figure 8, supports this simple logic.

9. The text will largely benefit with better definitions and accuracy:

a) SG formation is the consequence of eIF2alpha phosphorylation, as clearly stated on line 72. However, in several paragraphs this cause-consequence link is not that obvious and the way the effects are described suggests that SG formation and eIF2alpha phosphorylation are independent events or moreover, that eIF2alpha phosphorylation is the consequence of SG formation (line 449)!!!. I suggest to carefully revise the whole text regarding this.

b) Pro-survival and pro-death SGs (lines 145 and others). This is a vague concept. As stated in lines 51-52, the functions of SGs have yet to be elucidated, and if SGs are linked to cell death or cell survival is because they always form when cells are under stress. Stronger stress stimuli will induce higher levels of eIF2alpha phosphorylation, enhanced SG formation and higher levels of cell death.

c) Line 63: not only nuclear functions are represented in SGs: also RNA stability and translation regulation.

d) Line 68: the presence of 40S and translation initiation factors in SGs is the consequence of ribosome runoff.

e) Line 87: the info on HRI activation should be updated. This kinase was recently shown by independent reports to be activated by mitochondrial-released peptides.

f) Line 133: when mentioning the connection of glucocorticoids with the stress response (line 133): are glucocorticoids enhancers or inhibitors of the stress response?

g) Line 479: what will be the mechanistic link between SGs and mitotic catastrophe?

h) Line 524, it’s proposed that persistent SGs induce cell death. What will be the mechanism? Or is rather than persistent cell stress leads to persistent SGs and increased cell death?

i) Page 13, top paragraph: another example of strongly overinterpreted observations: the absence of SGs in G3BP KO cells, which are more sensitive to VRB, does not directly imply that “VRB-induced SGs promote cell survival” (see point 6 above). G3BP1 is tumorigenic and the multiple mechanisms involved were recently reviewed (DOI: 10.1080/1061186X.2018.1523415). That G3BP1 absence correlates with reduced cell viability is expected, is not a novelty per se and not necessarily linked to SG disruption.

j) Finally, the introduction and discussion sections altogether contain more text than the Results Section (9 pages vs 8 pages). In addition, the Results Section includes several introductory paragraphs that repeat the info described in the Introduction. Thus, is plenty of space to include substantial experimental work.

Reviewer 3

Advance summary and potential significance to field

In this work, authors have analyzed the effect of glucocorticoids with chemotherapy on SG formation and cell survival. They show that such co-treatment promote transition of pro-survival Sgs into pro-death. These findings provide new insights into molecular mechanisms to manipulate cancer cell chemosensitivity.

Comments for the author

I only have few issues to be addressed here before consideration for publication upon revision (Minor revisions)

© 2022. Published by The Company of Biologists under the terms of the Creative Commons Attribution License (https://creativecommons.org/licenses/by/4.0/).

Major:
It was shown that pro-death SGs are lacking specific protein components , and specifically subumits of eIF3 initiation factor, when compared with canonical prosurvival. Authors should determine if this is the case here

Minor:
1) Are other glucocorticoids besides cortisone have similar effects on VRB/SG/survival?
2)Would pre-treatment with cortisone before VRB change kinetics/ number of SGs when compared with co-treatment?
3) Would VRB or cortisone cause formation of ROS species that indirectly trigger eIF2alpha phosphorylation?
4) I would suggest authors to change term "rigid SGs". Technically, they have not directly analyzed physical properties of SGs or LLPS here...
5) Authors should also discuss possible heterogeneity of SGs in terms of RNA content (e.g., as in Advani VM CMLS 2020 on SG subtypes) upon VRB versus VRB+ cortisone treatment

---

First revision

Author response to reviewers' comments

We thank the reviewers for their insightful comments and suggestions. We have made a large effort to address all issues and hope the reviewers will feel, as we do, that the manuscript has substantially improved in light of their remarks.

Since one of the reviewers felt that the main conclusions were not clear we briefly reiterate the main findings of the study:

Glucocorticoids augment SG formation when administered in combination with the chemotherapies tested here. This was seen on a variety of levels and led to the following conclusions:

1. Imaging in living cells showed that cortisone increased SG assembly rates, however, the rates of SG disassembly after the stress were reduced, demonstrating lower clearance of the granules from the treated cells when cortisone was present. This formation of an altered SG structure was due to increased residence times of proteins within the SGs, whereas the dynamics of an mRNA in the SGs remained slow and unchanged. The modified biophysical properties of SGs due to cortisone may connect to enhancement of cell death.

2. Our study is the first to specifically show SG formation in human-derived organoids. Since Vinorelbine is administered to patients with metastatic breast cancer, we examined SG formation in organoids from healthy breast tissue and from metastatic breast tissue. Strikingly, Vinorelbine and cortisone induced SGs only in the diseased tissue and not in the healthy tissues.

3. SG formation coincides with the activation of the integrated stress response (ISR) pathway through the phosphorylation of eIF2α. We examined the signal transduction pathways activated by cortisone and Vinorelbine and discovered for cortisone, using kinase specific inhibitors and ISRIB, that it activates the GCN2 kinase of the ISR, hence the accumulative effect of cortisone when added together with the chemotherapy.

4. The population of cells containing SGs increased dramatically when cortisone was added to Vinorelbine. This was seen in human and mouse cells. Surprisingly, cortisone on its own, induced SG formation in mouse cells. This unique property of cortisone in murine cells helped us to reveal the kinase that cortisone activates within the integrated stress response.

5. Cortisone drove the complete cell population to more rapid cell death dynamics when added to Vinorelbine. We also found how this was accomplished. Typically, Vinorelbine as a microtubule destabilizer, interferes with cell proliferation and causes many cells to linger in an incomplete cell

© 2022. Published by The Company of Biologists under the terms of the Creative Commons Attribution License (https://creativecommons.org/licenses/by/4.0/).

division state that is termed mitotic catastrophe. Cortisone seems to eliminate this phase and this might promote cell death.

6. In ΔΔG3BP1/2 cells that cannot form SGs under VRB conditions there was little mitotic catastrophe. The addition of cortisone to ΔΔG3BP1/2 cells, led to an increase in cell survival compared to Vinorelbine alone. The phenotypes were rescued by the reintroduction of G3BP1. This can suggest that the lack of G3BP1/2 and SGs in conjunction with cortisone, protects cells from Vinorelbine- mediated cell death.
To accommodate the length guidelines and also following the comments by one of the reviewers regarding the length, we shortened the manuscript text.
We are sorry for the delay in response. We have made several attempts to collect more data on organoids. Growing them takes a very long time and we were only successful with another organoid from healthy tissue. Also, unfortunately, the primary author of the manuscript and her kids caught COVID in a chain reaction which hampered the flow of experiments.

Reviewer 1

- If feasible, it would be interesting to investigate how eIF2a abundance and phosphorylation is affected in non-malignant vs. patient-derived breast cancer organoids. This may provide insights on the discussed influence of eIF2a abundance on malignant transformation or therapy resistance, respectively.

Moreover, can the authors provide statistics on SG formation in organoids (Fig.3)?
A main constraint in working with organoids, is the that we are limited by the ability to grow large numbers of organoids. This caused very low throughput in the Western blotting in which we tried do perform examination of eIF2 phosphorylation, as suggested by the reviewer, which is indeed an area of interest for us. The time it took to obtain enough material was very long and the amount of protein extracted was very low so in the end we did not manage to detect the proteins. As part of this effort, we did try to obtain and grow additional types of organoids and on the way found that in another healthy breast tissue organoid there were no SGs formed upon VRB. We did not manage to grow additional types of cancerous organoids.

Regarding the statistics, we used a total of 30 organoid samples for each treatment and 3 biological replicates. Under arsenite treatment, 100% of cells were positive for stress granules in both cancerous and non-cancerous organoids. For both VRB and VRB+Cor treatments, 100% of the cancerous cells were positive for SGs, while 0% of the non-cancerous group were positive for SGs. We added the information in the Methods section and in Fig. S1.

Can the authors address or speculate why cortisol shows potentially distinct effects on SG dynamics in distinct cells, in particular in primary cancer organoids. May this be associated with eIF2a abundance or upstream signaling components?

We show that the glucocorticoids contribute to the ISR translation inhibition pathways already activated by the vinca alkaloids by activating another arm of the ISR. Other studies have shown that glucocorticoids repress initiation of mRNA translation by the assembly of the eIF4F complex and not a change in eIF2α phosphorylation or eIF2B activity (PMID: 10644539). So, it seems we have identified a new arm of glucocorticoid activation of the ISR via eIF2α that occurs in human cell lines and cancer organoids only when the pathway is first activated or primed by the vinca alkaloids, since cortisone alone does not activate the ISR at all or very little in the human cell lines we tested. Indeed, two organoid types from healthy patients did not form SGs and so we would speculate as the reviewer suggests that there might be increased eIF2α abundance or sensitivity in the cancer cells. It has been shown that malignant cells have high levels of these translation initiation factors (PMID: 29153484).

- Can the authors address or speculate (beyond rigidity) on the mechanisms behind the distinct protein dynamics observed in cortisol-induced SGs and why more rigid SGs may promote cell death?

© 2022. Published by The Company of Biologists under the terms of the Creative Commons Attribution License (https://creativecommons.org/licenses/by/4.0/).

To try address this question, we performed Western blot experiments for both G3BP1 and eIF4B to see if we could observe any mobility shifts that could indicate post-translational modifications such as phosphorylation of the proteins, however, none were observed for these molecules and might be relevant to others we did not check. We think that by the addition of the new FRAP experiments we have a strong indication that protein exchange in the granule is an important component. In particular, we performed FRAP on the IGF2BP3 protein in SGs in cells where the treatment had been rinsed, hypothesizing that the VRB+Cor treated SGs would have a similar recovery curve to those of the rinse, because the SGs were still present and exhibited impaired biophysical properties. In contrast, SGs in the cells treated with VRB only, should have a very different recovery curve. This was because the SGs are in the process of dispersing very quickly, and therefore they would recover less in the FRAP analysis; i.e. less protein will be re-entering in, if on a whole the proteins are on their way out of the structure as it dissolves. This was indeed the case. The VRB+Cor curve had a slight drop in overall recovery compared to the rinse that was not statistically significant (Fig. 4E, right). We predict that this slight drop is due to the fact some dissolution is taking place with these SGs as well, though significantly less in the 30 min post rinse when we FRAPed the SGs. However, the VRB rinsed cells had a significant drop in recovery (p value 0.0044 **), from ~70% to only about ~50% (Fig. 4E, middle). This then flips the curve; whereas before the curves showed less recovery for the VRB+Cor treated cells, now in the rinsed cells it had significantly more recovery due to the rapid dispersal of the VRB induced SGs. It is therefore possible to speculate that the impairment in dynamics negatively influences the balance of free proteins in the cytoplasm thereby preventing them from taking part in normal cell processes and not allowing translation to properly resume after the dissipation of the stress. Hence, the continued effects of the stress despite the removal of the stressor, and the continued presence of the SGs finally directs to cell death.

- most studies were performed at VRB concentrations ~100-fold higher than expected/reported (https://www.cancerrxgene.org/) IC50 values for the respective cell lines. Do the cells form SGs when treated for longer time (72h) at low doses of VRB +/- cortisol?

When establishing the desired concentration for treatment, we consulted Szaflarski et al, 2016 (PMID 27083003), which established that the IC50 of VRB in U2OS cells is 70 µM. We now mention this in the Methods. In the experiments of longer duration, we chose to use a less toxic dose of VRB (10 µM) in order not to immediately kill the cells, so to observe the changes in effect with and without the addition of cortisone. When we looked at 72 hrs we did not see SGs but studies have shown that they can form and then dissolve. The viability tests show that VRB at 10 µM has a cytotoxic effect over time.

- Minor typo: L65, instead of IGFBPs this should read IGF2BPs

Corrected.

- The authors should check if the used cell lines are indicated in each figure legend to support data evaluation.

Thanks for this comment. We checked the figure legends accordingly.

Reviewer 2

1. QUANTIFICATIONS IN Figure 1A, 1B, S4, S5, S6, S7 and S8 Showing a single image for each condition/staining is not acceptable. SG formation should be analyzed in a significant number of cells in several replicate. (Data in panel 1A can be omitted without substantial loss as this is not a novel observation and is repeated in other figures)

We thank the reviewer for pointing this out, this is indeed necessary, and we have added quantifications for the appropriate figures. We followed the presentation of this type of data as in Szaflarski et al, 2016 (PMID 27083003). We agree that panel 1A shows known observations and not all are needed as implied by the reviewer. Still, we feel it is important to briefly introduce the typical staining of cells under the VRB treatment in the opening figure, before turning to the organoids, especially for the readers that are not acquainted with this topic or for those who do not have the patience to go to look at the supplemental figures. Therefore, we reduced the data shown and only

© 2022. Published by The Company of Biologists under the terms of the Creative Commons Attribution License (https://creativecommons.org/licenses/by/4.0/).

present a small panel of staining patterns.
In Fig.1B- We quantified 30 organoids under each treatment – Fig. S1.

In Fig. 2, in addition to the quantifications we performed for Fig. 2B, we now added quantifications from the frames of the time-lapse videos under VRB treatment, and with the addition of Cor, as suggested by the reviewer. To test the number of SG-positive cells we performed quantification for 20 time points over an hour. The quantifications were performed on ~30 different videos and then averaged (Fig. 2F).
In Fig. 4D we quantified the time-lapse videos under VRB wash treatment separately, and with the addition of Cor. To test the number of SG-positive cells we performed quantification for 20 time points over an hour. The quantifications were performed on ~30 different videos and then averaged.
In Fig. S3 we collected the figures of the various cell types into one cohesive figure, and for each cell type we performed quantification for the number of cells that are positive for SGs

2. FIGURE 1A:

a) eIF3B is known to be mostly cytoplasmic in U2OS cells. Is there a reason for the strong nuclear signal shown here? b) P-2alpha: its presence in puncta is not clear. Is this a bonafide SG marker?
To re-examine this point raised by the reviewer we purchased a new antibody to eIF3B and the staining is cytoplasmic (see below figure for reviewer). For P-2alpha we agree that the staining was unclear, and since we are limited in the number of supplemental figures we decided only to show G3BP1, TIA1 and eIF4B in Fig. 1A, as mentioned above.

© 2022. Published by The Company of Biologists under the terms of the Creative Commons Attribution License (https://creativecommons.org/licenses/by/4.0/).

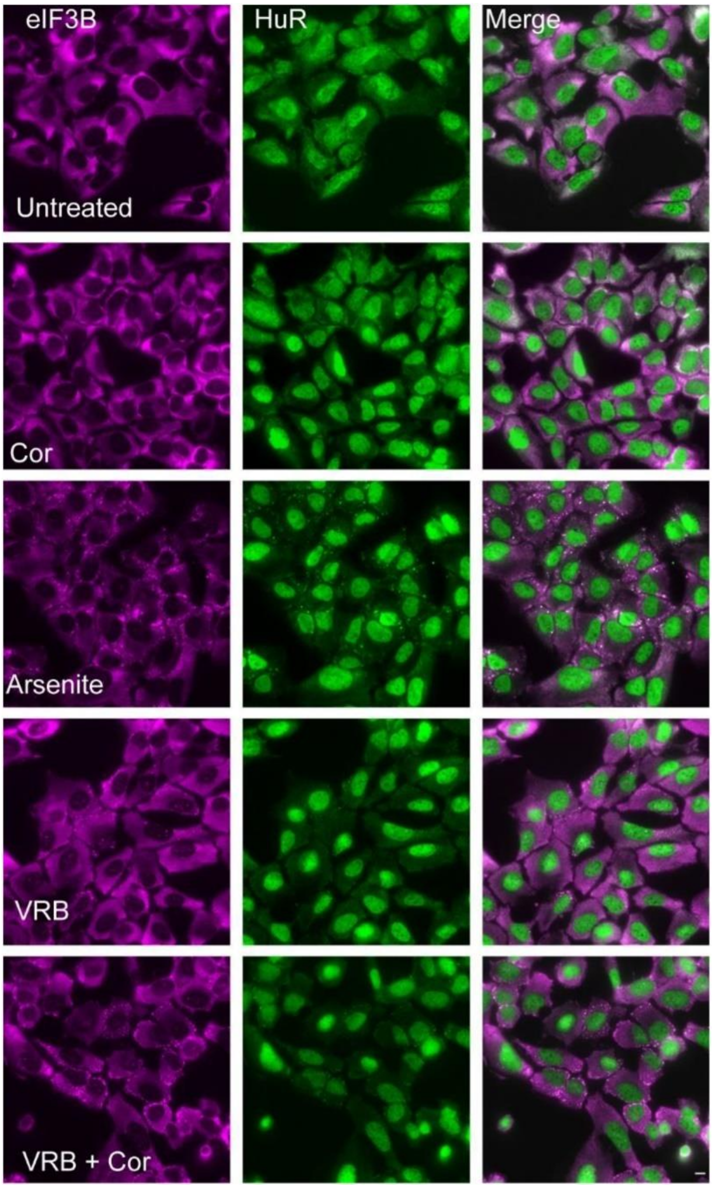

3. <u>Figure 1B</u>

a) As above, quantifications are strictly required. Showing a single example for each condition is not acceptable.
How many healthy and tumor-derived organoids were analyzed, and how many cells in each sample?

Regarding the statistics for the organoids, we now present the information in the Methods section and Fig. S1. We used a total of 30 organoid samples for each treatment and 3 biological replicates. Under arsenite treatment, 100% of cells were positive for stress granules in both cancerous and non-cancerous organoids. For both VRB and VRB+Cor treatments, 100% of the cancerous cells were positive for SGs, while 0% of the non-cancerous group were positive for SGs.

Were patients under any kind of treatment? Healthy and tumoral tissue from the same patient will work better.

The tumoral tissue was from a breast metastasis from the left hip, and the tumor mass from the muscle. The cells are ER-, PR-, HER2+. The patient was pretreated with Tamoxifen, Letrozol, Olaparib. The normal tissue was untreated. We tried to obtain additional organoids but this is challenging due to restrictions and regulations. We did manage to get another set of healthy breast

© 2022. Published by The Company of Biologists under the terms of the Creative Commons Attribution License (https://creativecommons.org/licenses/by/4.0/).

tissue organoids (BR63N). In the second healthy breast cells we also did not see the formation of SGs by VRB (figure for reviewer below).

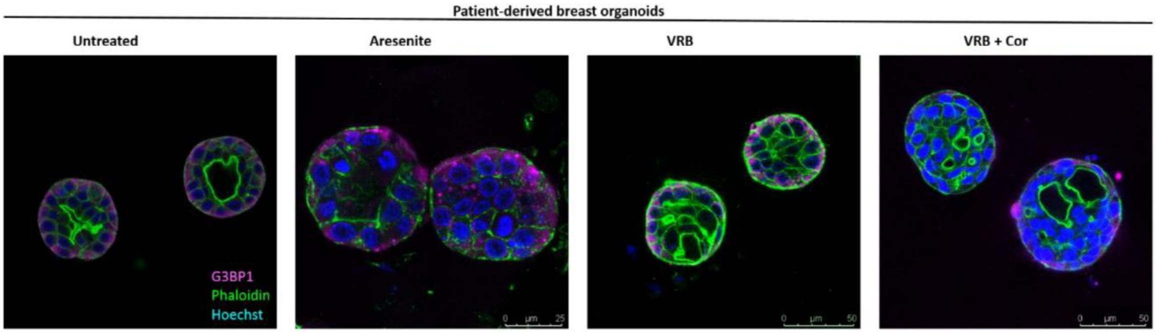

b) Line 178 and 222: data certainly do not show “the potency of VRB action on cancer cells”

We removed these statements.

4. <u>FIGURE 2</u>

a) FIGURE 2: In the “100 uM VRB plus Cor” panel, SGs are not apparent in the enlarged cell.

We redid the figure such that the granules can be properly seen.

b) In addition to the number of cells with SGs, quantification of the number of SG per cell and their size may help to describe the response. For example, SG seems larger upon 75uM VRB plus Cor than upon 75uM VRB alone. This may be in connection with a stronger ISR and stronger eIF2alpha phosphporylation.

This is indeed a valid point that we actually looked into when we started to examine the effects of cortisone on SG formation. However, after performing these experiments several times, we concluded that there was no obvious difference in the number or the size of the granules within a given cell. What was evident, is that there is a difference in the number of cells that are positive for stress granules across different treated populations, and for this we performed quantifications for cells in all conditions (Fig. 2B). As it pertains to quantifications, we used other publications as guidance and followed the practice of listing “% of cells with SGs” or “SG positive cells” as in Szaflarski et al, 2016 (PMID 27083003) or “foci positive cells” as in Aulas et al 2017 (PMID 28096475) in order to understand the effect on SG broadly in the population. Below we will elaborate on another change that occurs to the SGs upon addition to cortisone which could describe the response.

c) line 187: I agree that cell detachment is a sign of cell damage, suggesting that the insult is not a sublethal stress (see point 7 below).

d) Figure 2D and E, and Figure S9 (videos). SG formation and dissolution should be quantitated at several time points and independent replicates should be analyzed. Again, the number of cells with SGs is a binary readout that limits the analysis.

This was a good suggestion and new imaging and quantifications were performed on the processes of SG formation and dissolution at several time points, and independent replicates were analyzed (Fig. 4). We also show the kinetics in a graphical form. As listed above, the accepted metric of “SG positive cells” was used.

e) The initial distribution of IGF2BP3 in the cells shown in S9 is not comparable. The lower cell shows a greater intensity ratio SGs/cytosol, and it is expected that it will take longer for this signal to dissipate. See point d above.

To address this point, we examined and quantified multiple replicates to achieve robust statistical significance between the 2 treatment groups. As for the figure, we chose new fields to represent the dissolution effect, each with similar SG/cytosol ratio, and also present the analysis as a graph.

© 2022. Published by The Company of Biologists under the terms of the Creative Commons Attribution License (https://creativecommons.org/licenses/by/4.0/).

We now think that this data is important for presentation in the main figures (Fig. 4C), as part of the additional analysis we did on the FRAP experiments as encouraged by the reviewer, to be explained below.

f) Finally and very important, observations in 2D and E and S9 are redundant with the above results: if more cells show SGs at 60 min (panel 2B) is expected that more cells will show SGs at earlier time points (30 min) and that more cells will show SGs at later times, during recovery. And all this correlates with enhanced 2alphaP, as expected. It would be more clear if all these observations are presented as readouts of the same effect, rather than as independent effects of the treatment.

We thank the reviewer for encouraging us to think how to better present the kinetic results as a whole, and more importantly how to interpret and explain their logic. After the additional experiments we performed, we think we now have a plausible explanation regarding the dynamics of SG protein in the SGs under the different conditions.

To explore the phenomenon of impaired SG clearance after VRB+Cortisone treatment, we followed the number of SG positive cells after a rinse with fresh medium in both VRB and VRB+Cor treated cells in live-cell movies. Nearly all SGs are rinsed rapidly in VRB treated cells by ~40 minutes, while 70% of SG positive cells in VRB+Cor treatment persisted even one hour after rinse (Fig. 4C).

In order to visualize these data from a different angle, we looked at SG positive cells in the different treatment groups and considered that starting number as 100% positive. We chose to only consider positive cells in this metric because we wouldn't expect a SG-positive cell's SG clearance to be dictated by whether or not a neighboring cell contained SGs. When looking at the data in this manner, VRB treated cells drop from 100% positive to SGs to nearly 0, while VRB+Cortisone treated cells maintain SGs in about 70% of cells (Fig. 4D).

We then connect these live-cell experiments to the FRAP experiments. In the manuscript we show that for GFP-IGF2BP3 and G3BP1-GFP, the cortisone augmentation to VRB treatment caused less exchange of proteins in the SG (Fig. 4E,F, left). This matches the observation regarding the impaired clearance of the SGs after the treatment had been removed.

To buttress this thought, we performed FRAP on the IGF2BP3 protein in SGs in cells where the treatment had been rinsed. We hypothesized that the VRB+Cor treated SGs would have a similar recovery curve to those of the rinse, because the SGs <u>were still present</u> and exhibited impaired biophysical properties. In contrast, SGs in the cells treated with VRB only, should have a very different recovery curve. This was because the SGs <u>are in the process of</u> <u>dispersing very quickly</u>, and therefore they would recover less in the FRAP analysis; i.e. less protein will be re-entering in, if on a whole the proteins are on their way out of the structure as it dissolves. This was indeed the case. The VRB+Cor curve had a slight drop in overall recovery compared to the rinse that was not statistically significant (Fig. 4E, right). We predict that this slight drop is due to the fact some dissolution is taking place with these SGs as well, though significantly less in the 30 min post rinse when we FRAPed the SGs. However, the VRB rinsed cells had a significant drop in recovery (p value 0.0044 **), from ~70% to only about ~50% (Fig. 4E, middle). This then flips the curve; whereas before the curves showed less recovery for the VRB+Cor treated cells, now in the rinsed cells it had significantly more recovery due to the rapid dispersal of the VRB induced SGs.

As mentioned above we did not observe a noticeable change in SG size or abundance in cells that are positive for SGs, rather, there is a significant increase in SG positive cells after the addition of cortisone, as discussed above. The radical divergence in clearance times between VRB treatment with and without Cortisone is not only statistically significant but biologically relevant.

5. <u>**FIGURE 4:**</u> what do we learn from this? What is the relevance of describing the effect in mouse cells? Another example of descriptive work that lack mechanistic insights.

We think that these data were not in the right position in the sequence of logic of the manuscript and hence their relevance was not clear. In mouse cells we saw the formation of stress granules, under the administration of cortisone only (no VRB needed). This subsequently led us to better understand the mechanism since we could later test what signaling pathway is induced by the

© 2022. Published by The Company of Biologists under the terms of the Creative Commons Attribution License (https://creativecommons.org/licenses/by/4.0/).

cortisone without the background of VRB. We moved this figure downstream in manuscript to appear before examination of the mechanism.

6. **FIGURE 5F:** G3BP1 FRAP. G3BP1 is a key SG assembly factor and condense in the so-called SG cores. Thus, it is expected that G3BP1 exchange with the cytosol will be affected by the strength of the stress and the concomitant increase in SG formation (see point 3.e above). In other words, how specific is the observed effect for the VRB plus COR treatment? Is the nature of the stressor or the strength of the ISR the factor that affect SG dynamics? Does it change along the time-course of SG formation? Does it depend on the SG size? Is this change linked to the 2alpha kinase that is being activated (cortisone induces GCN2 and VRB induces PERK)? Investigating some of these questions will help understanding the reason of the observed change. Finally, does it make sense that mRNA exchange is not altered and at the same time protein exchange is affected? It will be nice to elaborate on this.

It was suggested by the reviewer that we should examine if the lowered recovery of G3BP1 in VRB+Cor was not due to a cortisone specific phenomenon, but rather because more G3BP1 was found in the core of more SGs, and therefore stronger stress would dictate slower recovery of the protein. This was a good idea and to ascertain whether this was the case and examine the specificity of the Cortisone treatment, we performed FRAP on SGs that had been treated with 0.5 mM of arsenite for 1 hr before treatment. This treatment of course is known to cause the formation of a high number of SGs and is considered a hallmark SG inducer and was used as a potent control in this study. In this case, the FRAP recovery curve was nearly identical to that of the VRB and not statistically significantly different (Fig 4F, right). Therefore, this strengthens our interpretation that there is indeed a significant difference between the VRB and VRB+Cor treatments owed to biophysical properties. Both the arsenite and VRB had nearly 100% recovery, while the VRB+Cor only reached about 80%. This then implies that G3BP1 exchanges rapidly in most SGs, but in VRB+Cor SGs it exchanges less due to the nature of the granule.

As for the mRNA, we only tested one type and there are definitely more species inside the SGs. This is indeed interesting compared to the protein exchange and would suggest that the mRNA component is more stable and that the exchange is occurring on the proteins that bind the mRNAs depending on their dynamics and available binding sites. As we cited in the article, it is known that different mRNAs show different residence times in SGs, it is possible this effect is seen more profoundly on an mRNA that would have more interactions with the sequestered protein.

7. The use of G3BP1/2 KO cells: The effect of the recovery G3BP1/2 levels should be analyzed. KO cells should be transfected with G3BP 1/2 and all measurements repeated. Phenotype rescue is an important control strictly required to conclude that the effects reported are specifically due to lack of G3BPs.

We thank the reviewer for this suggestion. We obtained the KO and KO cells rescued by G3BP1 cells form the Hornstein group and performed annexin and mitotic catastrophe experiments again with U2OS, G3BP1/2 KO and rescued cells. As seen in Fig. S7, U2OS and G3BP1-rescued cells showed a similar percentage of positive cells for mitotic catastrophe. In the annexin analysis, there was a significant difference between KO and rescued cells under VRB+Cortisone conditions. The addition of cortisone to the rescued cells did not trigger cell survival as was seen in the KO cells. We performed the annexin experiments under 48 hrs, which gave us the opportunity to compare VRB and VRB+ Cortsione treatments significantly.

8. The main conclusion: Authors propose that cortisone changes the biophysical properties of VRB-induced SGs and that this change the function of SGs from a protective role to a cell-death inducing role. This is not an immediate conclusion. Increased cell death may be linked to the stronger insult that results from the combined treatment. VRB activates the PERK pathway (how?) and cortisone activates GCN2 (how?). When both 2alpha kinases are activated the response is stronger, shifting from a sub-lethal to a more lethal stress. P-2alpha levels are higher (which is known to induce pro-apoptotic factors, CHOP among others) and SG formation is enhanced (with several parameters being affected, including reduced dynamics of SG core proteins). The data are insufficient to conclude that VRB-induced SGs are protective whereas SGs induced by VRB + cortisone are pro-death. Rather, SGs are a consequence and a marker of the cell reaction and the survival/death balance is not directly influenced by them. The effect of ISRIB shown here, which

© 2022. Published by The Company of Biologists under the terms of the Creative Commons Attribution License (https://creativecommons.org/licenses/by/4.0/).

inhibits the translational silencing downstream of P-2alpha and concomitant SG formation, as clearly summarized in figure 8, supports this simple logic.

We thank the reviewer for this interpretation, namely, that increased cell death may result from the combined activation of the two arms of the ISR. As we explained in Fig. 8 when trying to unravel the mechanism, we found that cortisone induced eIF2a phosphorylation by activating GCN2 which is different than what is known for VRB that activates PERK. Indeed, this explanation is plausible, and we now have added it to the discussion and interpretation of the results. Regarding the terminology of pro-death/survival, we have agreed that this should be rephrased as mentioned below.

9. The text will largely benefit with better definitions and accuracy:
a) SG formation is the consequence of eIF2alpha phosphorylation, as clearly stated on line 72. However, in several paragraphs this cause-consequence link is not that obvious and the way the effects are described suggests that SG formation and eIF2alpha phosphorylation are independent events or moreover, that eIF2alpha phosphorylation is the consequence of SG formation (line 449)!!!. I suggest to carefully revise the whole text regarding this.

We thank the reviewer for pointing this out and have corrected throughout the manuscript.

b) Pro-survival and pro-death SGs (lines 145 and others). This is a vague concept. As stated in lines 51-52, the functions of SGs have yet to be elucidated, and if SGs are linked to cell death or cell survival is because they always form when cells are under stress. Stronger stress stimuli will induce higher levels of eIF2alpha phosphorylation, enhanced SG formation and higher levels of cell death.

We agree that the difference between “pro death” and “pro survival” stress granules has not been fully clarified. We therefore have altered the text to reflect this, namely using the terminology “linked to cell death” rather than implicating a causal relationship. In this regard, we rely heavily on broad research linking SGs with impaired clearance to different pathologies and cell death.

c) Line 63: not only nuclear functions are represented in SGs: also RNA stability and translation regulation.

Corrected.

d) Line 68: the presence of 40S and translation initiation factors in SGs is the consequence of ribosome runoff.

Corrected.

e) Line 87: the info on HRI activation should be updated. This kinase was recently shown by independent reports to be activated by mitochondrial- released peptides.

Information was updated.

f) Line 133: when mentioning the connection of glucocorticoids with the stress response (line 133): are glucocorticoids enhancers or inhibitors of the stress response?

We added a citation of study showing that Glucocorticoids can cause oxidative stress.

g) Line 479: what will be the mechanistic link between SGs and mitotic catastrophe?

The aim was to show that cortisone has an effect not only on SG formation and cell death kinetics, but also played a role in the mechanism in which the cells die. We mention in the discussion that we speculate that mitotic catastrophe is connected to cell death kinetics, which might be influenced by the presence of SGs and G3BP1/2 proteins. Furthermore, after repeating the experiments with the G3BP1-rescued cells, we saw that the rescue of G3BP1 in the KO cells also rescued mitotic catastrophe in VRB treated cells, similarly to U2OS naïve cells (Fig. S7A-B). Therefore, we speculate that G3BP1 has an effect in the cell death mechanism under the conditions

© 2022. Published by The Company of Biologists under the terms of the Creative Commons Attribution License (https://creativecommons.org/licenses/by/4.0/).

of VRB, which might be the link between SGs and mitotic catastrophe.

h) Line 524, it's proposed that persistent SGs induce cell death. What will be the mechanism? Or is rather than persistent cell stress leads to persistent SGs and increased cell death?

As mentioned above to reviewer 1, the previous data in the manuscript and the new experiments done indicate impaired clearance in SGs induced by VRB and Cortisone. It is therefore possible to speculate that the impairment in dynamics negatively influences the balance of free proteins in the cytoplasm thereby preventing them from taking part in normal cell processes and not allowing translation to properly resume after the dissipation of the stress. Hence, the continued effects of the stress despite the removal of the stressor, and the continued presence of the SGs finally directs to cell death.

i) Page 13, top paragraph: another example of strongly overinterpreted observations: the absence of SGs in G3BP KO cells, which are more sensitive to VRB, does not directly imply that "VRB-induced SGs promote cell survival" (see point 6 above). G3BP1 is tumorigenic and the multiple mechanisms involved were recently reviewed (DOI: **10.**1080/1061186X.2018.1523415). That G3BP1 absence correlates with reduced cell viability is expected, is not a novelty per se and not necessarily linked to SG disruption.

Indeed, as pointed out by the reviewer, despite its role in SG biology, G3BP1 is involved in various mechanisms that are linked to cancer and cell viability. We do not overrule the possibility that the effect of G3BP1 on cell death is not limited to the absence of SGs in the KO cells. We thank the reviewer for this important observation and therefore added this clarification to the discussion. It is logical that G3BP1 plays a role in cell death in terms of SGs, since it was shown previously that there is a direct correlation between SG formation and cell viability specifically under VRB treatment. This was demonstrated by the depletion of G3BP1 and G3BP2 in U2OS cells (Szaflarski et al. 2016).

j) Finally, the introduction and discussion sections altogether contain more text than the Results Section (9 pages vs 8 pages). In addition, the Results Section includes several introductory paragraphs that repeat the info described in the Introduction. Thus, is plenty of space to include substantial experimental work.

We revised the text as suggested.

Reviewer 3

I only have few issues to be addressed here before consideration for publication upon revision (Minor revisions).

Major:
It was shown that pro-death SGs are lacking specific protein components , and specifically subumits of eIF3 initiation factor, when compared with canonical prosurvival. Authors should determine if this is the case here.
We saw an accumulation of the protein eIF3 under the administration of VRB, however, we did not see a decrease in protein levels under the administration of VRB and Cor together. We suggest that cortisone-induced SGs are linked to cell death in terms of biophysical properties. With regard to the SG definition, since we have had comments about this terminology, we now use a different definition of pro-death/survival not based on components but on biophysics. Also, since pro-death/ survival is a controversial topic, we have decided to change the terms pro-death/survival to the terminology "linked to cell death".

Minor:
1) Are other glucocorticoids besides cortisone have similar effects on VRB/SG/survival?

We thank the reviewer for this suggestion. We explored co-treating VRB with other glucocorticoids as well. The glucocorticoid Prednisone showed similar effects to that of the cortisone, and when VRB was co-treated with Prednisone we saw the same raise in SG positive cells. This appears now in Fig. S4.

© 2022. Published by The Company of Biologists under the terms of the Creative Commons Attribution License (https://creativecommons.org/licenses/by/4.0/).

2) Would pre-treatment with cortisone before VRB change kinetics/ number of SGs when compared with co-treatment?

Thanks for this suggestion. We explored the potential differential treatment between pre-treating cells with cortisone vs co-treatment. There was no difference in the number of SG positive cells when cortisone was used before the administration of the VRB. See figure below for the reviewer.

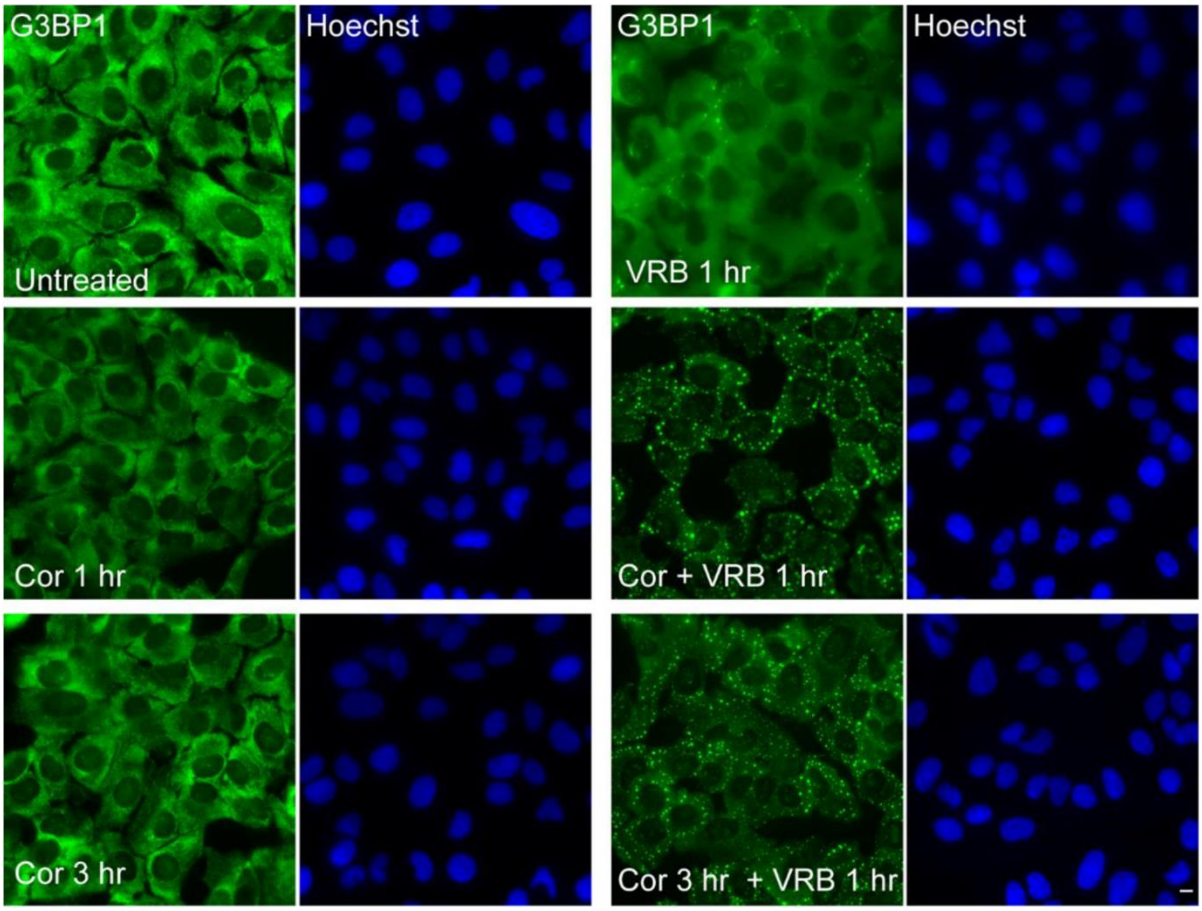

3) Would VRB or cortisone cause formation of ROS species that indirectly trigger eIF2alpha phosphorylation?

There are a few articles showing that VRB or glucocorticoids can cause oxidative stress via ROS (PMIDs: 29113247, 22030390, 31216749) but we didn't explore this pathway in this study. We mention this option in the Discussion.

4) I would suggest authors to change term "rigid SGs". Technically, they have not directly analyzed physical properties of SGs or LLPS here...

We accept this comment and have changed accordingly.

5) Authors should also discuss possible heterogeneity of SGs in terms of RNA content (e.g., as in Advani VM CMLS 2020 on SG subtypes) upon VRB versus VRB+ cortisone treatment

We note this important point and this also encouraged us to examine a variety of RNAs using RNA FISH, including mRNA and lncRNA, and indeed we could observe transcripts docking on both types of SGs, but did not observe any significant difference in composition between the two treatment groups. This data was added to the manuscript.

© 2022. Published by The Company of Biologists under the terms of the Creative Commons Attribution License (https://creativecommons.org/licenses/by/4.0/).

Second decision letter

MS ID#: JOCES/2021/259629

MS TITLE: Glucocorticoids enhance chemotherapy-driven stress granule assembly and impair granule dynamics leading to cell death

AUTHORS: Avital Schwed-Gross, Hila Hamiel, Gabriel P Faber, Mor Angel, Rakefet Ben-Yishay, Jennifer Israel-Cohen, Dana Ishay-Ronen, and Yaron Shav-Tal
ARTICLE TYPE: Research Article

I am happy to tell you that your manuscript has been accepted for publication in Journal of Cell Science, pending standard ethics checks.

Reviewer 1

Advance summary and potential significance to field

In their study Schwed-Gross et al. investigate the dynamic formation of stress granules (SGs) and SG-association with stress signaling pathways as well as cell viability upon treatment with cortisol and/or chemotherapeutics of the vinca-alkaloid family, specifically vinorelbine (VRB). The authors present very interesting findings, including one of the first investigations of these compounds in cancer organoids.
Findings by Schwed-Gross et al., reveal that VRB- and cortisol-dependent induction of SGs is eIF2a-dependent, but relies on partially distinct upstream signaling, PERK- vs. GCN2-modulated pathways. Most interestingly, the authors show a strong cell type-dependence of their findings and distinct protein/RNA-dynamics in/at SGs upon VRB/cortisol treatment. The study is of particular interest for the cancer field, since SG formation was observed exclusively in patient-derived breast cancer organoids, but not non-malignant breast organoids. Although the study remains largely descriptive it provides highly valuable information for further exploration of the role(s) SGs may serve in the clinical management of cancer by chemotherapy. The presented manuscript is well-written, although the authors may want to consider shortening the introduction and discussion where appropriate. In sum, the presented study provides valuable, high-quality insights which set the stage to further explore the highly complex role of SGs and G3BPs in diseases, in particular cancer.

Comments for the author

The authors have addressed all my (minor) and other previous concerns.

Reviewer 2

Advance summary and potential significance to field

As mentioned previously, the work correlates changes on eIF2alpha phosphorylation, SG formation and cell survival upon a combination of drugs of clinical relevance.

Comments for the author

I found the work greatly improved and congratulate the authors for the additional analysis performed. However, a quite important issue remains and it is the sometimes defective logic in a number of sentences, as I have indicated before. For example, the title states that “glucocorticoids enhance chemotherapy-driven stress granule assembly and impair granule dynamics leading to cell death”. Certainly, the work does not include evidence that impaired granule dynamics is causative of cell death. The title MUST reflect conclusions supported by experimental evidence.
I would like to emphasize that the MS does not benefit at all with this kind of overstatements. Experiments are neatly performed and stating cause-consequences relationships that are not

© 2022. Published by The Company of Biologists under the terms of the Creative Commons Attribution License (https://creativecommons.org/licenses/by/4.0/).

supported by a clear logic certainly doesn´t increase the overall value of the work. It rather compromises its significance.
Also, please check all along the manuscript that the text is clear in that 2alpha phosphorylation precedes SGs formation.

Reviewer 3

Advance summary and potential significance to field

All my issues were addressed adequately. I can now recommend the work for publication

Comments for the author

No issues

© 2022. Published by The Company of Biologists under the terms of the Creative Commons Attribution License (https://creativecommons.org/licenses/by/4.0/).
